# Cysteine restriction‐specific effects of sulfur amino acid restriction on lipid metabolism

**DOI:** 10.1111/acel.13739

**Published:** 2022-11-19

**Authors:** Sailendra N. Nichenametla, Dwight A. L. Mattocks, Diana Cooke, Vishal Midya, Virginia L. Malloy, Wilfredo Mansilla, Bente Øvrebø, Cheryl Turner, Nasser E. Bastani, Jitka Sokolová, Markéta Pavlíková, John P. Richie, Anna K. Shoveller, Helga Refsum, Thomas Olsen, Kathrine J. Vinknes, Viktor Kožich, Gene P. Ables

**Affiliations:** ^1^ Animal Science Laboratory Orentreich Foundation for the Advancement of Science Cold Spring‐on‐Hudson New York USA; ^2^ Department of Environmental Medicine and Public Health Icahn School of Medicine at Mount Sinai New York New York USA; ^3^ Department of Animal Bioscience University of Guelph Guelph Ontario Canada; ^4^ Department of Nutrition, Institute of Basic Medical Sciences University of Oslo Oslo Norway; ^5^ Department of Pharmacology University of Oxford Oxford UK; ^6^ Department of Pediatrics and Inherited Metabolic Disorders, General University Hospital in Prague Charles University‐First Faculty of Medicine Prague Czech Republic; ^7^ Department of Probability and Mathematical Statistics Charles University ‐ Faculty of Mathematics and Physics Prague Czech Republic; ^8^ Departments of Public Health Sciences and Pharmacology Penn State University College of Medicine Hershey Pennsylvania USA

**Keywords:** aging, caloric restriction, cysteine, metabolic syndrome, methionine, nutrition, sulfur amino acids, triglycerides

## Abstract

Decreasing the dietary intake of methionine exerts robust anti‐adiposity effects in rodents but modest effects in humans. Since cysteine can be synthesized from methionine, animal diets are formulated by decreasing methionine and eliminating cysteine. Such diets exert both methionine restriction (MR) and cysteine restriction (CR), that is, sulfur amino acid restriction (SAAR). Contrarily, SAAR diets formulated for human consumption included cysteine, and thus might have exerted only MR. Epidemiological studies positively correlate body adiposity with plasma cysteine but not methionine, suggesting that CR, but not MR, is responsible for the anti‐adiposity effects of SAAR. Whether this is true, and, if so, the underlying mechanisms are unknown. Using methionine‐ and cysteine‐titrated diets, we demonstrate that the anti‐adiposity effects of SAAR are due to CR. Data indicate that CR increases serinogenesis (serine biosynthesis from non‐glucose substrates) by diverting substrates from glyceroneogenesis, which is essential for fatty acid reesterification and triglyceride synthesis. Molecular data suggest that CR depletes hepatic glutathione and induces Nrf2 and its downstream targets Phgdh (the serine biosynthetic enzyme) and Pepck‐M. In mice, the magnitude of SAAR‐induced changes in molecular markers depended on dietary fat concentration (60% fat >10% fat), sex (males > females), and age‐at‐onset (young > adult). Our findings are translationally relevant as we found negative and positive correlations of plasma serine and cysteine, respectively, with triglycerides and metabolic syndrome criteria in a cross‐sectional epidemiological study. Controlled feeding of low‐SAA, high‐polyunsaturated fatty acid diets increased plasma serine in humans. Serinogenesis might be a target for treating hypertriglyceridemia.

## Graphical abstract

The proposed mechanism of CR‐specific effects of SAAR on adipose metabolism. (1) Due to the lack of Cys in SAAR diets, it imposes both MR and CR. CR specifically results in decreased biosynthesis of the tripeptide glutathione. (2) Lower hepatic glutathione increases the abundance of the transcription factor Nrf2, which translocates to the nucleus and induces the transcription of *Phgdh.* (3) Increase in the transcription and translation of *Phgdh* and *Pck‐2* results in higher hepatic serinogenesis (Ser biosynthesis from substrates other than glucose, that is, from oxaloacetic acid). (4) Higher serinogenesis competes with glyceroneogenesis as both pathways use the same set of substrates, which results in lower fatty acid re‐esterification. *Note*: broken arrows represent the presence of other biochemical intermediates not shown in the figure. Up and down arrows adjacent to the metabolic intermediates represent an increase and decrease, respectively. 3PG, 3‐phosphoglycerate; OAA, oxaloacetic acid; DHAP, dihydroxyacetone phosphate; PEP, phosphoenolpyruvate; FA, fatty acids.

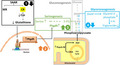

## 1 INTRODUCTION

Dysregulated lipid metabolism is a common etiological factor in obesity, metabolic syndrome (MetS), and type 2 diabetes (Stout et al., 2017). It also increases the risk for inflammatory diseases and cancers (Catalan et al., 2013; Gutierrez et al., 2009). Improved lipid metabolic homeostasis not only alleviates these diseases but also extends overall healthspan. Sulfur amino acid restriction (SAAR), lowering the intake of methionine (Met) in the absence of cysteine (Cys), improved lipid metabolism in rodents, even with ad libitum access to the diet (Zhou et al., 2016). SAAR conferred resistance to diet‐induced obesity and induced weight loss in obese mice within 2 weeks (Ables et al., 2012; Cooke et al., 2020). SAAR‐induced metabolic benefits include decreases in body weight, fat mass, plasma triglycerides, and leptin, and increases in plasma adiponectin and fibroblast growth factor 21 (Fgf21). The high reproducibility of SAAR‐induced effects across multiple labs spurred clinical studies to combat obesity and MetS (Olsen et al., 2018, 2020; Plaisance et al., 2011). However, the few clinical studies conducted show that its impact on humans is modest compared to that in non‐human animals. The molecular signature of SAAR, including altered expression of hormones (insulin‐like growth factor 1 [Igf1], Fgf21, adiponectin, and leptin), enzymes (fatty acid synthase, acetyl‐CoA carboxylase, and stearoyl‐CoA desaturase), and metabolites (S‐adenosylmethionine and S‐adenosylhomocysteine) is well‐characterized in rodents. But the biochemical events that drive these changes remain unknown (Zhou et al., 2016). Understanding the biochemistry of SAAR‐induced changes in rodents is critical for its translation to humans.

Met and Cys are the two proteinogenic sulfur amino acids (SAA) with distinct biological roles. Although the metabolic requirement of Cys is essential, its dietary requirement is dispensable, as healthy adult humans and non‐human animals can convert Met to Cys through the transsulfuration pathway (Womack et al., 1937). Accordingly, the nutritional requirement of total SAA (Met and Cys together), in synthetic rodent diets, are usually satisfied by providing only Met. Most animal studies formulated SAAR diets by decreasing the Met content by approximately 80% (control diets: 0.86% w/w dry matter content; SAAR diets: 0.12%–0.17% w/w dry matter content) and eliminating Cys. This approach is justified if the objective is to investigate the combined effect of low SAA on a response variable but not their discrete effects. Although healthy rodents have a well‐functioning transsulfuration pathway, Met concentration in the SAAR diet is too low to meet growth and metabolic demands for Cys (Reeves et al., 1993). This insufficiency implies that rodents on SAAR undergo both Met restriction (MR) and Cys restriction (CR). Whether the SAAR‐induced changes are due to the combined effect or discrete effects of MR and CR remains unknown. Understanding the discrete roles of MR and CR is critical as multiple epidemiological studies and a few laboratory studies suggest that the SAAR effects on lipid metabolism are due to CR but not MR.

While most laboratory studies eliminated Cys in SAAR diets, some supplemented it. In one study, when male F344 rats were fed a SAAR diet (0.17% w/w Met without Cys) supplemented with 0.5% w/w Cys, SAAR‐induced decreases in body weight, adipose depot weights, plasma triglycerides, leptin, total cholesterol, and LDL cholesterol were reversed (Elshorbagy et al., 2011). In another study, mature C3H/HeH mice were fed two diets, each with different Cys concentrations (0.07% w/w and 0.87% w/w) but with the same Met concentration (0.29% w/w). Mice on the high‐Cys diet had greater body weights, adipose depot weights, hepatic triglycerides, plasma triglycerides, and LDL cholesterol than those on the low‐Cys diet (Elshorbagy, Church, et al., 2012). Large‐scale epidemiological studies show that plasma Cys, but not Met, is positively associated with body mass index (BMI) and fat mass independent of caloric intake and physical activity, thus rendering support to the preclinical studies (Elshorbagy, Kozich, et al., 2012). Despite these data, one cannot ascribe changes in lipid metabolic markers to CR alone as the reversal of SAAR‐induced changes by Cys supplementation could be due to the biological abundance of Met caused by the sparing effect of dietary Cys on the transsulfuration pathway.

In the current study, we used depletion–repletion bioassays to demonstrate that MR and CR exert discrete effects on SAAR‐induced changes. Based on transcriptional and translational changes, we propose biochemical and molecular mechanisms for CR‐induced changes in lipid metabolism. We also investigate whether the CR‐specific effects depend on sex, age‐at‐onset, and dietary fat in C57BL/6J mice. Later, we test the relevance of our preclinical findings to humans by analyzing biospecimens and data from a previously conducted population study in individuals with different degrees of MetS. In addition, we reanalyze data from two short‐term studies in which participants were on low SAA diets.

## 2 RESULTS

### 2.1 Methionine restriction and cysteine restriction exert discrete effects on morphometry and plasma hormones

**FIGURE 1:**
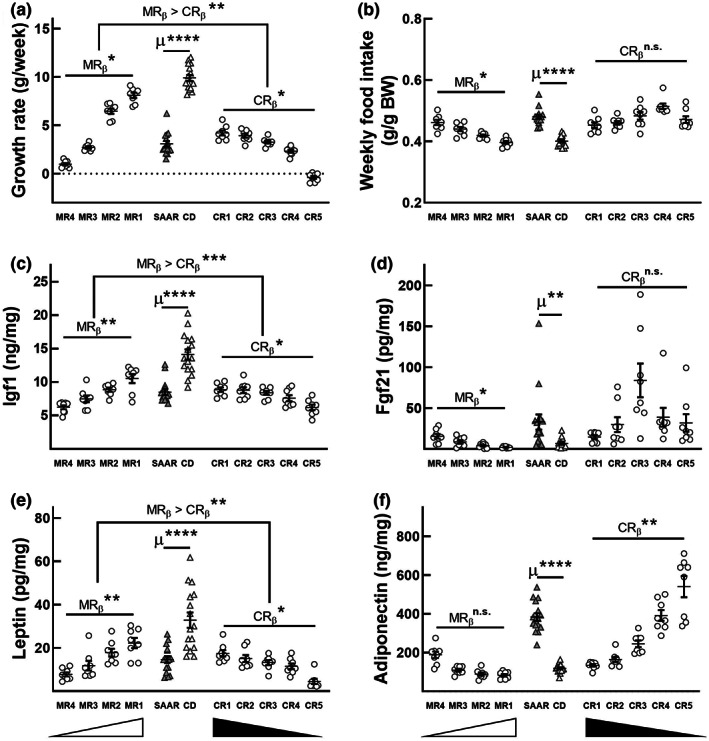
Methionine restriction and cysteine restriction exert discrete effects on morphometry and plasma hormone concentrations. Eight‐week‐old male F344 rats were fed CD, SAAR, MR (MR1, MR2, MR3, MR4), and CR (CR1, CR2, CR3, CR4, CR5) diets for 12 weeks. Changes in growth rate (a), food intake (b), plasma Igf1 (c), plasma Fgf21 (d), and plasma leptin (e) were dependent on MR, while changes in plasma adiponectin (f) were dependent on CR. Two‐tailed Student's *t*‐test was used to find differences (represented by μ) between CD and SAAR groups (*n* = 16). Dose responses to MR‐ and CR‐titrated diets were analyzed by simple linear regression (coefficients represented by MR_β_ and CR_β_; *n* = 8/group); error bars represent the means and standard error of means. *p < 0.05, **p < 0.01, ***p < 0.001, ****p < 0.0001, n.s. = not significant. White and black triangles below the *x*‐axes represent the SAA gradient in MR and CR diets, respectively

Due to the transsulfuration pathway, effectuating MR without CR or vice versa is not straightforward. To overcome this problem, we took a stepwise approach. First, to obviate the need for Met conversion to Cys, we fed a cohort of rats four MR diets, all replete with 0.5% w/w Cys but progressively restricted in Met (MR1: 0.17%, MR2: 0.10%, MR3: 0.07%, MR4: 0.05% w/w; Table S1a,b). Two control diets, CD (0.86% w/w Met without Cys) and SAAR (0.17% w/w Met without Cys), were also used. As observed in previous studies, SAAR decreased growth rates (Figure 1a, the ratio of mean values of SAAR to CD [SAAR/CD]: 0.31; *p* < 0.0001), Igf1 (Figure 1c, SAAR/CD: 0.60; *p* < 0.0001), and leptin (Figure 1e, SAAR/CD: 0.44; *p* < 0.0001), but increased food consumption (Figure 1b, SAAR/CD: 1.19; *p* < 0.0001), Fgf21 (Figure 1d, SAAR/CD: 4.86; *p* < 0.01), and adiponectin (Figure 1f, SAAR/CD: 3.23; *p* < 0.0001; Nichenametla et al., 2020).

To determine the discrete effects of MR, we investigated if Met‐titrated diets exerted a significant dose‐response on these phenotypes (MR_β_, regression coefficient of MR diets). Growth rates (Figure 1a, MR_β_ [−0.89]; *p* < 0.05), food intake (Figure 1b, MR_β_ [0.86]; *p* < 0.05), Igf1 (Figure 1c, MR_β_ [−0.59]; *p* < 0.01), Fgf21 (Figure 1d, MR_β_ [0.55]; *p* < 0.05), and leptin (Figure 1e, MR_β_ [−0.50]; *p* < 0.01) exhibited a strong dose‐response to MR, but adiponectin did not (Figure 1f). The concentration of Met in one of the four titrated diets that resulted in a similar effect size as the SAAR diet (0.17% Met without Cys) was considered as the dose of restriction required for the discrete effects of MR in the SAAR diet. Based on equivalence tests, we determined that this dose is 0.07% (MR3; Figure S1). In the second cohort of rats, the discrete effects of CR were determined by feeding five different diets, all consisting of 0.07% Met but progressively restricted in Cys (CR1: 0.5%, CR2: 0.25%, CR3: 0.12%, CR4: 0.06%, and CR5: 0.03% w/w). MR‐dependent markers were either non‐responsive (Figure 1b,d, food intake and Fgf21) or less responsive (Figure 1a,c,e, growth rate: CR_β_ [−0.34]; *p* < 0.05, Igf1: CR_β_ [−0.19]; *p* < 0.05, and leptin CR_β_ [−0.25]; *p* < 0.05) to CR than to MR. Plasma adiponectin, which did not respond to MR, exhibited a strong dose response to CR (Figure 1f, CR_β_ [0.62]; *p* < 0.01). Overall, our data confirm that among the changes induced by the SAAR diet, changes in growth rates, food intake, plasma Igf1, and leptin were MR‐dependent (Figure 1a–e). In contrast, plasma adiponectin was CR‐dependent (Figure 1f). Among the five CR diets, CR4 yielded a similar effect size for adiponectin as the SAAR diet (Figure S2).

An earlier study using the same concentrations of Met and Cys as in MR1 concluded that the reversal of MR‐induced changes was due to Cys supplementation (Elshorbagy et al., 2011). We now show that with progressive Met restriction, MR‐dependent changes recapitulate in MR2, MR3, and MR4 diets, even when supplemented with 0.5% Cys. This phenomenon indirectly proves that the reversal of certain SAAR‐induced changes observed in the previous report resulted from the sparing effect of dietary Cys on the transsulfuration pathway. Another report suggested that the ideal concentration range of Met in the SAAR diet was between 0.17% and 0.25% and that levels below 0.12% invoke amino acid deprivation responses (Forney et al., 2017). Although this interpretation is accurate, our findings show that when supplemented with 0.5% Cys, Met concentration can be lowered to 0.05% without any adverse effects (none of the diets used in this study induced amino acid deficiency signs).

### 2.2 Methionine restriction and cysteine restriction induce distinct changes in plasma amino acid profiles

**FIGURE 2:**
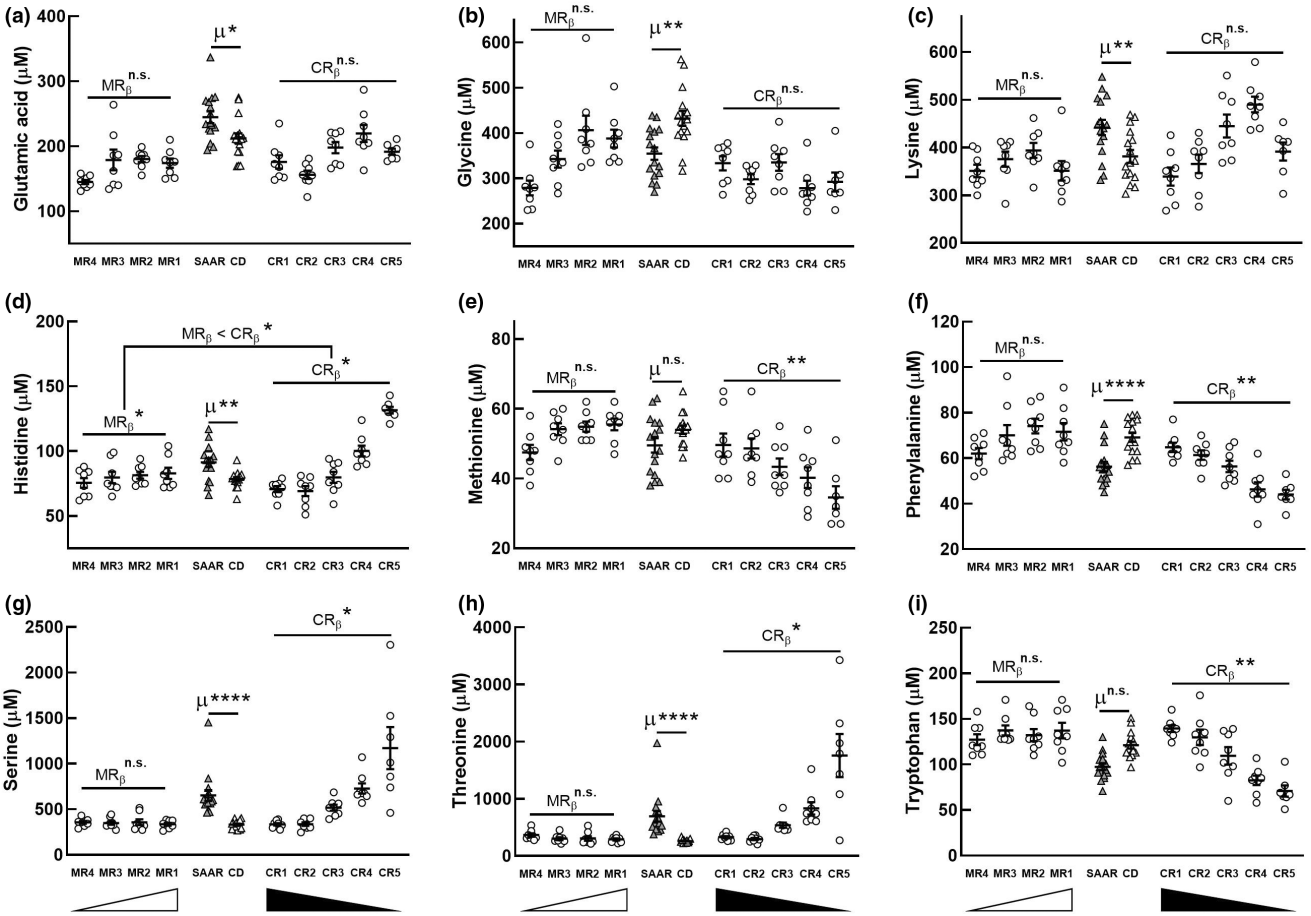
Methionine restriction and cysteine restriction induce distinct changes in plasma amino acid concentrations. Although SAAR changed plasma concentrations of glutamic acid (a), glycine (b), and lysine (c), these amino acids did not show dose response to either MR or CR. A strong dose response was exhibited by plasma histidine (d), methionine (e), phenylalanine (f), serine (g), threonine (h), and tryptophan (i) to CR. For sample sizes, statistics, and annotations refer to Figure 1

We previously reported that SAAR decreases hepatic protein synthesis rates by 33% (Nichenametla et al., 2018), but the metabolic fate of the amino acids not utilized for protein synthesis is unknown. After deamination/transamination, the carbon skeleton of amino acids can affect lipid metabolism through anaplerosis. We and others reported that individuals on a low Met diet had higher levels of plasma oxaloacetate, indicating changes in the TCA cycle (Gao et al., 2019; Martinez‐Reyes & Chandel, 2020). We quantified plasma amino acids to determine if they mediate some of the SAAR‐induced changes in adipose metabolism and to find if MR and CR discretely affect their concentrations. Of the 20 proteinogenic amino acids identified in our analysis, SAAR increased the levels of glutamic acid (ratio of the mean concentrations of SAAR to CD [SAAR/CD]: 1.15, *p* < 0.05), lysine (SAAR/CD: 1.16, *p* < 0.01), histidine (SAAR/CD: 1.16, *p* < 0.01), serine (Ser, SAAR/CD: 1.97, *p* < 0.0001), and threonine (SAAR/CD: 2.65, *p* < 0.0001), and decreased the levels of phenylalanine (SAAR/CD: 0.81, *p* < 0.0001) and glycine (SAAR/CD: 0.82, *p* < 0.01; Figure 2a–i). It is noteworthy that despite significant differences between CD and SAAR, glutamic acid, glycine, and lysine have not responded to either MR or CR (Figure 2a–c). On the other hand, phenylalanine (CR_β_ [−0.51]; *p* < 0.01), histidine (CR_β_ [0.69]; *p* < 0.05), Ser (CR_β_ [0.55]; *p* < 0.05), threonine (CR_β_ [0.54]; *p* < 0.05), and tryptophan (CR_β_ [−0.62]; *p* < 0.01) showed a dose response to CR but not to MR (Figure 2d–i). Despite not being different between CD and SAAR, Met exhibited a dose response to CR (Figure 2e, CR_β_ [−0.41], *p* < 0.01). Due to the technical difficulty of accurately quantifying Cys, we did not quantity it. Since Ser alters lipid metabolism markers, we focused our additional analyses on it (Esch et al., 2020; Gantner et al., 2019; Gao et al., 2018; Muthusamy et al., 2020).

### 2.3 Cysteine restriction, but not methionine restriction, induces hepatic de novo serine biosynthesis

**FIGURE 3:**
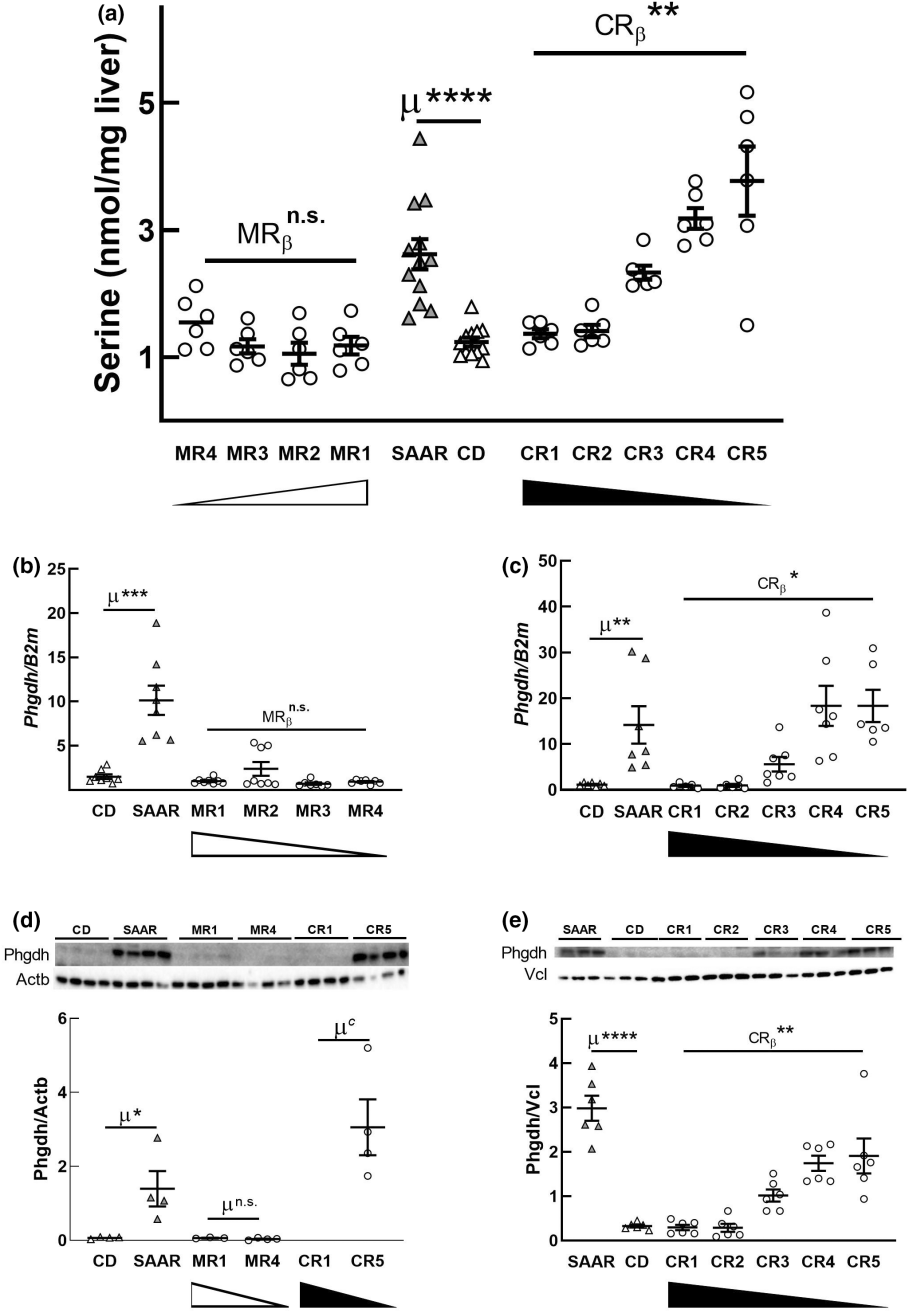
Cysteine restriction, but not methionine restriction, induces hepatic de novo serine biosynthesis. CR, but not MR, increased hepatic serine concentrations (a), *Phgdh* mRNA (b, c), and Phgdh protein levels (d, e). For statistics and annotations refer to Figure 1. In panel a, *n* = 16 for CD and SAAR groups, *n* = 8/group for all other groups; in all other panels, *n* = 4–8/group; in panel d, only the diets with the highest and lowest concentration of Met (MR1 and MR4) and Cys (CR1 and CR5) were probed. μ^c^—cannot determine (due to the absence of bands, numbers for band densities were not available)

To understand the role of Ser in lipid metabolism, we wanted to identify its source. Although the kidney and liver are the two major sources of plasma Ser, we quantified only hepatic Ser, as kidneys were not collected (Brosnan & Hall, 1989; Lowry et al., 1987). In agreement with a recent report, we found that SAAR caused a 2‐fold increase in hepatic Ser (Figure 3a, *p* < 0.0001; Stone et al., 2021). Similar to the CR‐dependent increase in plasma Ser, the increase in hepatic Ser was also dependent on CR (CR_β_ [0.59]; *p* < 0.01) but not on MR. To confirm if hepatic Ser increased due to its de novo biosynthesis, we quantified the mRNA levels of the rate‐limiting enzyme for Ser biosynthesis, phosphoglycerate dehydrogenase (Phgdh). Hepatic *Phgdh* expression was approximately 6‐ to 9‐fold higher in SAAR than in CD (Figure 3b,c, *p* < 0.01). A robust dose response was found in *Phgdh* levels with CR but not MR (Figure 3b,c, CR_β_ [0.51]; *p* < 0.05). Similar to our findings, previous studies based on nuclear run‐off assays show that dietary protein regulates *Phgdh* mRNA post‐transcriptionally. Higher Cys, but not Met, concentrations destabilized *Phgdh* mRNA (Achouri et al., 1999). To confirm whether mRNA increases reflect protein levels, we probed for hepatic Phgdh in rats fed diets with the highest and lowest SAA levels (Figure 3d, MR1, MR4, CR1, and CR5). MR1 and MR4 had low but similar levels of Phgdh. CR1 had no bands, but the band intensity in CR5 was greater than in SAAR. Similar to its effect on mRNA, CR exerted a dose response on Phgdh protein levels (Figure 3e, CR_β_ [0.43]; *p* < 0.01). These data demonstrate that the SAAR‐induced increase in de novo hepatic Ser biosynthesis is specifically due to CR.

### 2.4 3‐Phosphoglycerate, the substrate for serine biosynthesis, is derived from mitochondrial oxaloacetate

**FIGURE 4:**
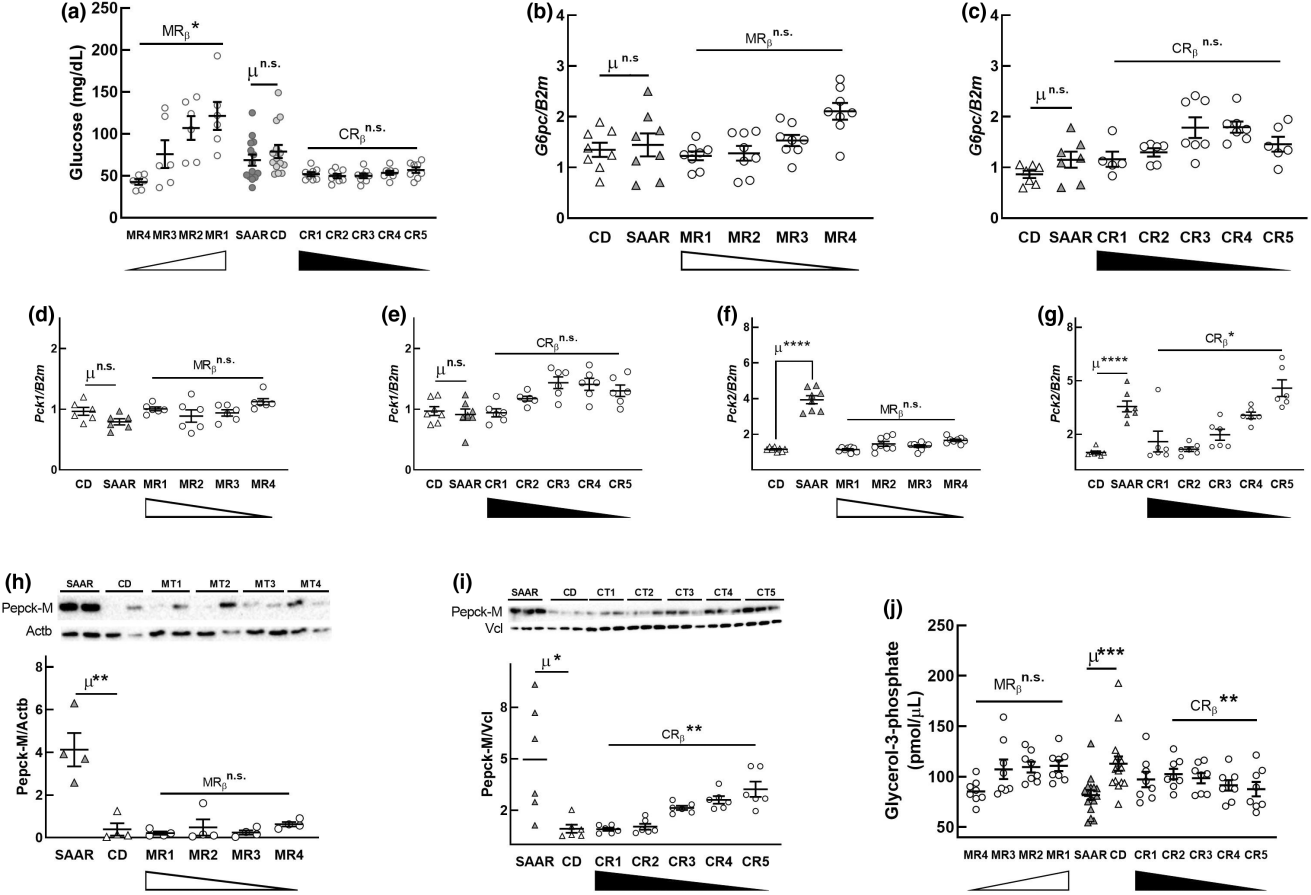
Cysteine restriction increases serinogenesis at the expense of glyceroneogenesis. MR, but not CR, decreased blood glucose (a). Neither MR nor CR altered the mRNA expression of *G6pc* (b, c) and *Pck1* (d, e). CR, but not MR, increased the hepatic mRNA expression of *Pck2* (f, g) and its protein Pepck‐M (h, i), and decreased hepatic glycerol‐3‐phosphate concentration (j). For statistics and annotations refer to Figure 1. In panel a, *n* = 14 for CD and SAAR groups, *n* = 8/group for all other groups; in all other panels, *n* = 4–8/group

To sustain the increase in hepatic de novo Ser biosynthesis, a constant supply of the substrate 3‐phosphoglycerate (3PG) is required. Under fed conditions, the canonical pathway for Ser biosynthesis is the “phosphorylated pathway,” in which the substrate 3PG is derived from glucose through glycolysis (Murtas et al., 2020). If CR‐induced Ser biosynthesis is through glycolysis, blood glucose might exhibit a dose‐dependent decrease. Our data show that, unlike plasma Ser, fasting blood glucose responded to MR but not CR (Figure 4a, MRβ [−0.68]; *p* < 0.5), which indirectly suggests that glycolysis is not contributing to Ser biosynthesis. Another likely reason for the non‐glycolytic origin of Ser is that hepatic glycolysis is minimal during fasting states; plasma and tissues were obtained after an overnight fast. Since 3PG can also be derived from gluconeogenesis, we probed for the mRNA expression of two enzymes that regulate this pathway, phosphoenolpyruvate carboxykinase‐C (Pepck‐c, coded by gene *Pck1*) and glucose‐6‐phosphatase‐C (G6pC, the catalytic subunit of G6p). SAAR did not alter either of these enzymes (Figure 4b–e), suggesting that gluconeogenesis may not be responsible for CR‐induced Ser biosynthesis. To the best of our knowledge, there is no other primary source of 3PG besides glycolysis and gluconeogenesis pathways.

Based on a recent report that the mitochondrial form of Pepck (Pepck‐M, coded by the gene *Pck2*) increased Ser biosynthesis, we quantified hepatic *Pck2* mRNA (Brown et al., 2016). We not only found that SAAR increased *Pck2* mRNA expression by at least 3.5‐fold (Figure 4f–g, *p* < 0.0001) but also that it exhibited dose response to CR (CR_β_ < 0.05) but not to MR. In addition, we also confirmed that the CR‐induced increase in the *Pck2* transcript is reflected in protein levels (Figure 4h–i; SAAR/CD: 5–10‐fold, *p* < 0.05 or lower) and that the changes in Pepck‐M are CR‐dependent (Figure 4i, CR_β_ < 0.01). These data indicate that CR increases the utilization of mitochondrial oxaloacetate to replenish cytosolic phosphoenolpyruvate, the substrate, and product of Pepck‐M, respectively. Based on these data, we infer that the pathway involved in CR‐induced Ser biosynthesis is serinogenesis (Ser biosynthesis from non‐glucose substrates) and not the typical phosphorylated pathway.

### 2.5 An increase in de novo serine biosynthesis depletes glycerol‐3‐phosphate required for glyceroneogenesis

The substrates and enzymes of the proximal gluconeogenesis pathway are shared by two other pathways, serinogenesis, and glyceroneogenesis. Increased consumption of substrates by one pathway can affect the end‐product concentration of the other. Recent studies demonstrate that higher PCK2 expression is associated with increased glyceride‐glycerol synthesis (Leithner et al., 2018). We asked if CR‐induced increases in Pepck‐M and serinogenesis affect gluconeogenesis and glyceroneogenesis. Although CR upregulated Pepck‐M (Figure 4i), it did not alter the mRNA levels of *G6pc* and blood glucose levels. This lack of effect suggests that CR does not alter gluconeogenesis. Our previous finding that SAAR does not change pyruvate tolerance in mice supports this interpretation (Ables et al., 2012). To determine whether CR is affecting glyceroneogenesis, we quantified glycerol‐3‐phosphate (G3P) and found that CR, but not MR, decreases hepatic G3P (Figure 4j, CR_β_ [−0.27 (CR2‐CR5)] < 0.01). Rats on SAAR had lower hepatic G3P concentrations than those on CD (Figure 4j, SAAR/CD: 0.72, *p* < 0.001). Earlier, it was reported that administering ethionine, the ethyl analog of Met and an inhibitor of Met‐dependent functions, including transsulfuration, decreased G3P concentration and thus lipogenesis in both liver and adipose tissue (Tani et al., 1973). Glyceroneogenesis provides a significant portion of glyceride‐glycerol required for the reesterification of fatty acids and triglyceride cycling (Nye et al., 2008). CR‐induced diversion of 3PG to serinogenesis from glyceroneogenesis might be the underlying mechanism for CR‐specific effects of SAAR on lipid metabolism.

### 2.6 The transcription factor, Nrf2, mediates cysteine restriction‐induced serinogenesis

Cys is a structural component of the tripeptide glutathione (GSH). We previously demonstrated that SAAR decreases hepatic non‐protein‐bound total GSH (tGSH = rGSH [GSH in reduced form] + GSSG [GSH in oxidized form]) and found a CR‐specific decrease in hepatic GSH of rats on Cys‐titrated diets (Figure S3; Nichenametla et al., 2018). Low GSH induces the transcription factor nuclear factor erythroid 2‐related factor 2 (Nrf2), which regulates Ser biosynthetic enzymes Phgdh, Psat, and Psp (DeNicola et al., 2015). Nrf2 expression is sensitive to dietary fat content, sex, and age (Yin et al., 2020; Zhang et al., 2015). We explored if SAAR induces GSH/Nrf2/Phgdh/Pepck‐M axis in the liver and if its activation depends on dietary fat content, sex, and age‐at‐onset. We fed 8‐week‐old male and female C57BL/6J mice CD and SAAR diets consisting of 10% Kcal or 60% Kcal fat for 4 months and 18‐month‐old male and female mice the 10% Kcal fat CD and SAAR diets for 3 months (Table S2).

**FIGURE 5:**
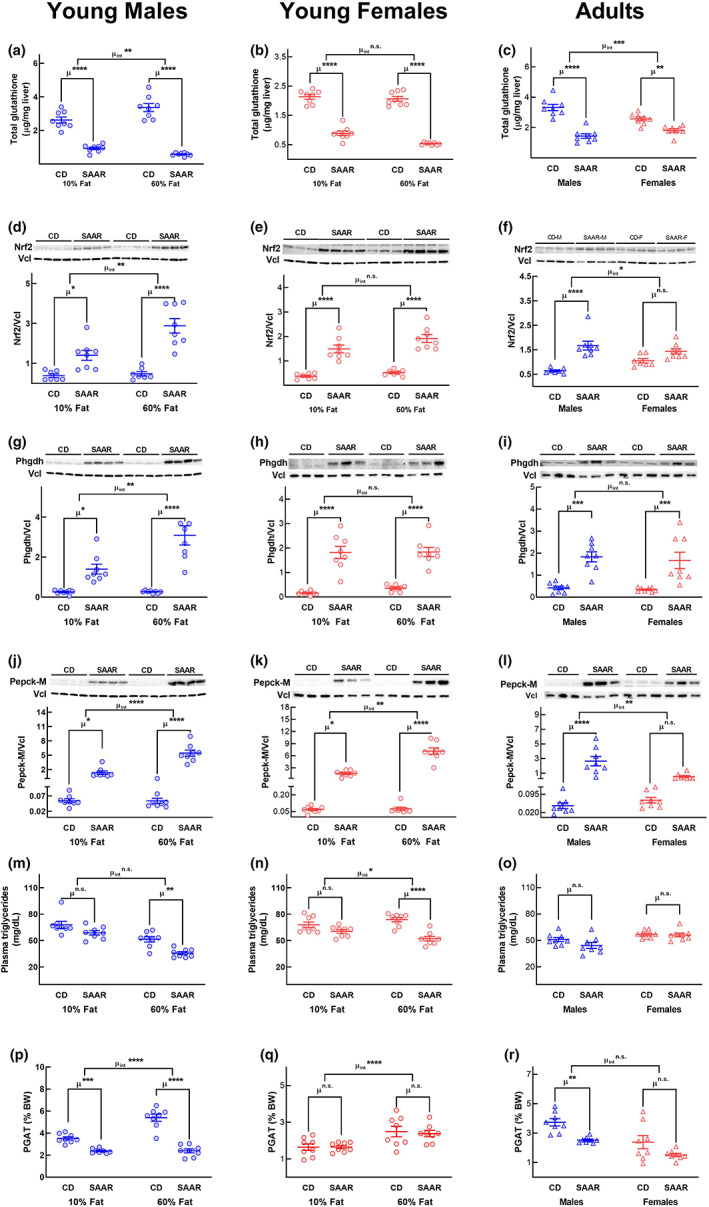
SAAR‐induced changes in molecular markers of serinogenesis and lipid metabolism are dependent on sex, dietary fat content, and age‐at‐onset. Young (8‐week‐old, circles) and adult (18‐month‐old, triangles) male (blue circles) and female (red circles) C57BL/6J mice were fed CD and SAAR diets with either 10% fat or 60% fat for at least 3 months. Statistical significance of SAAR‐induced changes in hepatic glutathione (a–c), hepatic protein expressions of Nrf2 (d–f), Phgdh (g–i), and Pck2 (j–l), plasma triglycerides (m–o), and perigonadal adipose tissue weights (p–r) were analyzed by 2‐way ANOVA. The interaction of SAAR‐induced changes with dietary fat in young mice and sex in adult mice are indicated by μ_int_. Pairwise comparisons between CD and SAAR are indicated by μ. *n* = 8/group; PGAT—perigonadal adipose tissue; error bars indicate means and standard error of means. *p < 0.05, **p < 0.01, ***p < 0.001, ****p < 0.0001, n.s. = not significant

Data from these three models were consistent with our hypothesis. SAAR decreased hepatic GSH, increased Nrf2 protein expression, and induced *Phgdh* and *Pck2* (Figure 5a–l). In young male mice, the effect size was generally greater on 60% fat than on 10% fat (Figure 5, column 1). In young females, changes were similar to those observed in males, but effect sizes were similar regardless of the dietary fat content, except for Pepck‐M (Figure 5, column 2). Sex did not alter the effect size on 10% fat diets. But, on a 60% fat diet, the effect sizes were significantly larger in males than in females (Table S3). In males, similar differences were found regardless of age‐at‐onset, but in females, a greater effect size was observed with young‐onset compared to adult‐onset (Table S4).

We also found a good agreement between the molecular markers and the ultimate phenotypes, that is, plasma triglycerides and adipose depot weights in males. Plasma triglycerides were significantly lower in young male and female mice fed SAAR on a 60% fat diet but not on a 10% fat diet than those fed control diets (Figure 5m–n). Triglyceride concentrations between CD and SAAR were not different in adult mice (Figure 5o). Perigonadal adipose tissue depot weights were significantly lower in male mice on SAAR regardless of age‐at‐onset and dietary fat content (Figure 5p–r) but not in female mice (Figure 5q). The differential activation of the GSH/Nrf2/Phgdh/Pepck‐M axis might underlie the dependency of SAAR‐induced changes on age‐at‐onset, sex, and dietary fat observed in previous studies (Forney et al., 2020; Nichenametla et al., 2020).

### 2.7 Plasma total cysteine and serine, but not methionine, correlate with triglyceride concentrations and metabolic syndrome criteria in humans

**FIGURE 6:**
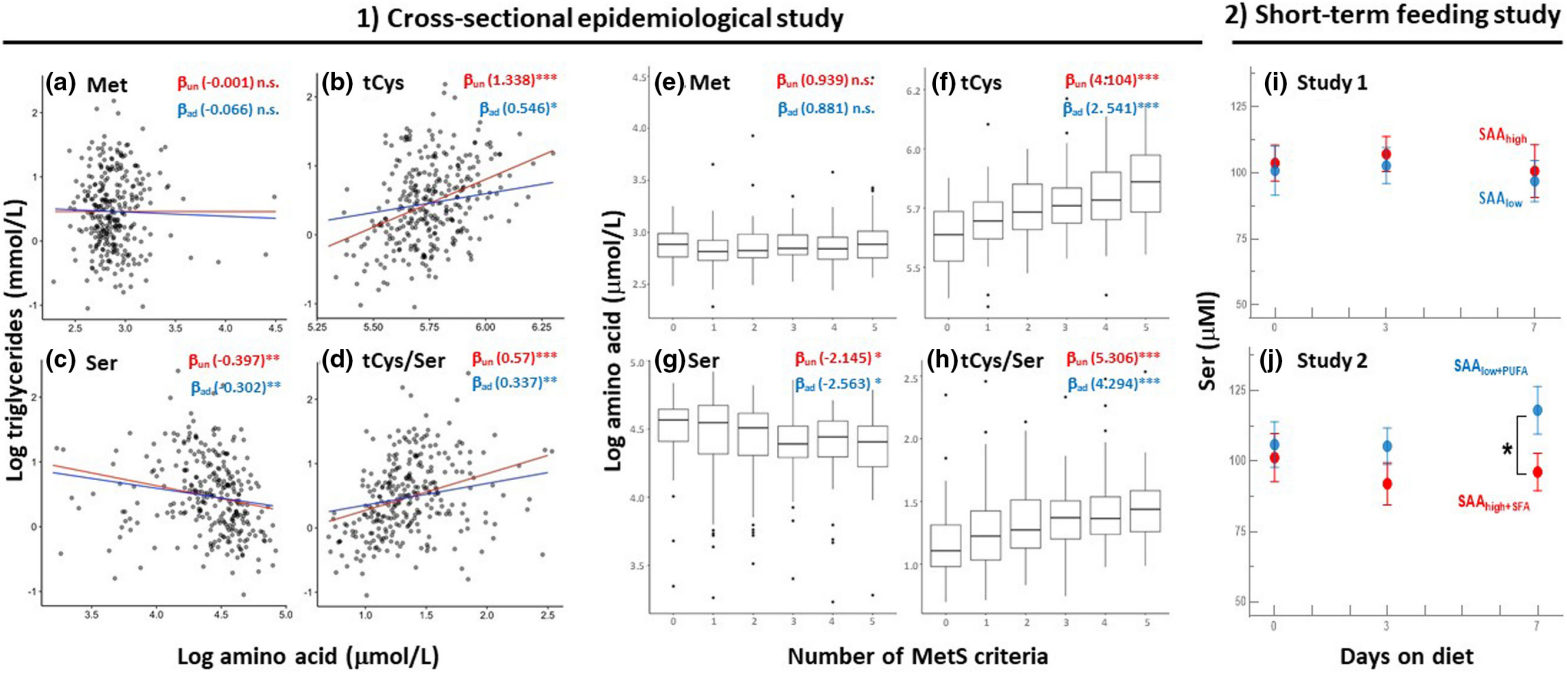
Associations between cysteine, serine, and triglycerides in epidemiological and short‐term human feeding studies reflect mechanistic findings from rodent studies. *1) Plasma tCys, Ser, and tCys/Ser correlate with plasma triglycerides and the number of MetS criteria in humans*. Log‐transformed plasma concentrations of Met (a, e), tCys (b, f), Ser (c, g), and tCys/Ser (d, h) were plotted against log‐transformed triglycerides (a–d) and MetS criteria (e–h), respectively. Red and blue lines in a‐d represent unadjusted and adjusted (for age, sex, and BMI) regression lines, respectively. *n* = 307 for plasma Met and tCys and *n* = 287 for plasma Ser; box plots represent the distribution of unadjusted amino acid concentrations within each category of MetS. Numbers in parenthesis after β_un_ and β_ad_ represent regression coefficients from unadjusted and adjusted models, respectively. (*p < 0.05, **p < 0.01, ***p < 0.001, ****p < 0.0001, n.s. = not significant). (2) Plasma Ser levels were amenable to low SAA diets depending on dietary fatty acid composition. (i) Change in plasma serine in overweight/obese women (*n* = 13) on a 7‐day pilot dietary intervention trial with low (SAA_low_) or high (SAA_high_) sulfur amino acid content. *p*‐Values represent group x time interactions (change in plasma serine over time between groups) and were computed with linear mixed models adjusted for baseline levels of serine. (b) Change in plasma serine in normal‐weight individuals (*n* = 14) participating in a 7‐day pilot dietary intervention trial with high polyunsaturated fatty acid contents and low SAA concentrations (SAA_low+PUFA_) versus a diet high in saturated fatty acids and high sulfur amino acids (SAA_high+SFA_). Red and blue dots represent mean Ser concentrations on high‐ and low‐SAA diets, respectively. Error bars represent confidence intervals

Association of plasma amino acids (Met, tCys [reduced Cys + oxidized Cys‐Cys + protein‐bound Cys], Ser, and tCys/Ser) with triglycerides and the occurrence of MetS criteria were investigated from a previously conducted epidemiological study (Janosikova et al., 2003). Descriptive statistics of subjects are provided in Table S5. Regression estimates and *p*‐values for associations of individual amino acids with triglycerides and MetS criteria are given in Figure 6a–h and Table S6. In models fully adjusted for age, sex, and BMI, triglycerides did not change with an increase in plasma Met (Figure 6a) but increased by 0.54% (Figure 6b, 95% CI: from 0.12 to 0.96, *p* < 0.05) with a 1% increase in plasma tCys and decreased by 0.30% (95% CI: from −0.49 to −0.10, *p* < 0.05) with a 1% increase in plasma Ser (Figure 6c). Triglycerides increased by 0.33% with a 1% increase in tCys/Ser (Figure 6d, 95% CI: from 0.15 to 0.52, *p* < 0.05).

Next, we questioned if plasma Met, tCys, Ser, and tCys/Ser were associated with the occurrence of MetS criteria. Plasma Met was not associated with MetS criteria (Figure 6e). In the unadjusted model, plasma tCys concentration increased by 4.10% (95% CI: from 3.14 to 5.07, *p* < 0.001) with an increase in each MetS criterion (Figure 6f). The estimate for tCys was attenuated but remained significant after adjusting for age and sex (2.54%, 95% CI: from 1.18 to 3.13, *p* < 0.001). Plasma Ser decreased with an increase in the number of MetS criteria in both models (unadjusted model, by −2.14%, 95% CI: from −4.12 to 0.12, *p* < 0.05, Figure 6g; adjusted model, by −2.56%, 95% CI: from −4.27 to −0.34, *p* < 0.05); however, the association of tCys/Ser with MetS criteria was more robust than the association with either amino acid alone. In the unadjusted model, the ratio of tCys/Ser increased by 5.31% with an increase in each MetS criteria (95% CI: from 3.08 to 7.57, *p* < 0.001, Figure 6h). After adjusting for age and sex, the tCys/Ser ratio increased by 4.29%, with increases in each MetS criteria (95% CI: from 1.86 to 6.78, *p* < 0.01). Overall, findings from the epidemiological study are consistent with mechanisms suggested by our animal data, that is, tCys deficiency results in higher Ser biosynthesis and lower triglycerides (Figure 6b,c). Data also show that the ratio of tCys/Ser might serve as a better biomarker for MetS than the individual amino acids.

### 2.8 Low‐SAA diets increase plasma serine depending on the dietary fatty acid composition

To test if low SAA diets alter plasma Ser levels, we used data from two previously conducted short‐term controlled feeding studies (Olsen et al., 2018, 2021). In Study 1, participants were fed diets with either low (SAA_low_) or high (SAA_high_) SAA (Table S7). In Study 2, to potentiate the effect of SAA_low_, the percent of polyunsaturated fatty acids was increased (SAA_low+PUFA_) while in SAA_high_, the percent of saturated fatty acids was increased (SAA_high+SFA_). Both studies were conducted for 1 week, and changes in plasma Ser over time (on days 0, 3, and 7) were compared between diet groups. Dietary composition and sample sizes are provided in Table S7.

Compared to plasma Ser on day 0, the estimated marginal mean Ser concentration on day 7 slightly decreased in both SAA_low_ (from 100.8 to 96.8 mmol/L) and SAA_high_ (from 103.7 to 100.7 mmol/L) groups in Study 1. The time‐related changes between the two diet groups were similar (Figure 6i and Table S8, *p*_int_ = 0.94). In Study 2, there was an increase in estimated marginal mean plasma Ser concentrations from 106 to 118 mmol/L in the SAA_low+PUFA_ group and a decrease from 101.2 to 96.1 in the SAA_high+SFA_ group; these time‐related changes between the two diet groups were significantly different (Figure 6j and Table S8, *p*_int_ <0.05). The associations between SAA, Ser, triglycerides, and MetS criteria in human studies are consistent with mechanistic findings from rodent studies.

## 3 DISCUSSION

Improvement of lipid metabolism by SAAR is well documented in rodent models, but mechanisms are unclear. To this end, we conducted dietary SAA depletion‐repletion bioassays. Data show that MR and CR exert discrete and non‐nutritive biological effects. MR‐dependent SAAR‐induced changes include decreases in growth rate, blood glucose, plasma Igf1, and leptin, and increases in food consumption and Fgf21 (Figures 1 and 4a). CR‐dependent SAAR‐induced changes include increases in plasma adiponectin, Ser, threonine, and histidine and decreases in plasma Met, tryptophan, and phenylalanine (Figures 1f and 2). Molecular data show that CR decreases hepatic tGSH (Figure S3) and induces Nrf‐2 and its downstream effectors, Phgdh and Pepck‐M, which increase serinogenesis. The simultaneous CR‐induced decrease in G3P and increase in serinogenesis indicate that higher serinogenesis depletes the substrates required for glyceroneogenesis. Since glyceroneogenesis provides most of the glyceride‐glycerol required for the reesterification of fatty acids and triglyceride synthesis, its decrease might eventually manifest as lower adipose depot weights. We also show that the SAAR‐induced changes in molecular markers are consistent and reproducible in three experimental models. Effect size varied with age‐at‐onset (young > adult), sex (male > female), and dietary fat (60% fat > 10% fat). Consistent with data from rodent models, we found that plasma Ser negatively correlated with plasma triglycerides and MetS criteria in humans, while plasma Cys positively correlated with both. We also show that plasma Ser might be amenable to low SAA diets in humans when combined with high polyunsaturated fatty acids.

Our current understanding of the roles Cys plays in energy metabolism is relatively less than that of its functions associated with thiol‐disulfide exchanges. In addition to affecting energy metabolism by contributing to its carbon moiety, Cys acts as a signaling molecule. Cys contributed up to 10% of intracellular pyruvate in pancreatic cancer cells and up to 20% upon knocking out pyruvate kinase (Yu et al., 2019). Cys also affects energy metabolism through mTOR signaling (Gu et al., 2017). Our data suggest that Cys plays a much broader role in regulating central carbon metabolism than merely contributing to its carbon skeleton. By depleting GSH, CR induced Pepck‐M, which can alter the TCA cycle. Phgdh, the second CR‐induced enzyme, is typically associated with glycolysis (Locasale et al., 2011). However, our data imply that it could also play a role in glyceroneogenesis. Through its thiol‐disulfide function, Cys can increase adiponectin secretion and affect adipose metabolism (Lee & Shao, 2014). In vitro studies demonstrate that intracellular retention and secretion of adiponectin depend on thiol concentrations (Wang et al., 2007). We speculate that the increased adiponectin secretion in our study could be due to lower glutathione concentration in the liver and adipose tissues. Overall, our data provide a mechanistic basis for the effects of Cys on metabolic health. The nutritional requirements of Met and Cys, for both laboratory animals and humans, are always considered together. But, three diets with different ratios of Cys to Met (0.2, 1, and 2), despite having the same total SAA concentration, had different effects on body weight, blood urea nitrogen, and bile acids in rats (Sarwar et al., 1991). From a disease prevention perspective, our data show that considering individual dietary intakes of Met and Cys is crucial.

Unlike the well‐studied effects of inhibiting Ser biosynthesis on cancers, only a few studies have investigated its role in lipid metabolism (Muthusamy et al., 2020). Our data suggest that serinogenesis lowers glyceroneogenesis by limiting the availability of G3P (Figure 4j). Other studies prove the corollary, that is, inhibition of Phgdh decreased plasma Ser and increased hepatic fat content in mice fed Met‐choline‐deficient diets (Sim et al., 2020). Of note, the composition of the Met‐choline‐deficient diet is different from that of the SAAR diet. The former is deficient in methyl donors, that is, both Met and choline are low; hepatic steatosis occurs due to inadequate VLDL secretion (Marcolin et al., 2011). Although the SAAR diet is low in Met, it is not deficient in methyl donors due to abundant choline. Unlike Met‐choline‐deficient diets, SAAR decreases hepatic steatosis (Malloy et al., 2013). In addition, Phgdh transgenic mice on a high‐fat diet had lower hepatic triglycerides than wild‐type mice, indicating that an increase in Ser biosynthesis, independent of the CR diet, can affect lipid metabolism (Sim et al., 2020). The epidemiological association of *PHGDH* DNA methylation with triglycerides suggests that Ser might play a similar role in lipid metabolism in humans and non‐human animals (Truong et al., 2017). This finding is consistent with results from our epidemiological study, where plasma Ser was inversely associated with triglycerides and MetS criteria (Figure 6). Another epidemiological study documented significantly lower Ser in diabetic patients than in controls (Zhou et al., 2013). We propose serinogenesis as an excellent target in treating hypertriglyceridemia and associated lipid metabolism disorders.

Our data do not explain if increased Ser has any functional implications. Ser supplementation increased yeast lifespan (Enriquez‐Hesles et al., 2021). Its ability to support one‐carbon metabolism through methyl group donation is thought to play a role. Considering the lifespan extension by SAAR, one could expect a similar function. But, it is less likely that Ser plays such a role as the SAAR diet has other fungible methyl donors: choline and glycine (Table S1a). Ser can increase endogenous Cys biosynthesis by condensing with homocysteine. This reaction also commits the S atom in homocysteine to Cys rather than to Met (through remethylation). Since the SAAR diet lacks Cys, higher Ser to support Cys biosynthesis might seem plausible. We doubt if this is the case, as SAAR decreases hepatic homocysteine (Tamanna et al., 2018). Stoichiometrically, increasing serine without a concomitant increase in homocysteine would not increase Cys biosynthesis. While we did not quantify hepatic homocysteine in our study, other studies show that it does not change in rats on a SAAR diet (Tamanna et al., 2018). On the other hand, homocysteine export was reported to be sensitive to Ser availability and the ratio of S‐adenosylmethionine to S‐adenosylhomocysteine (Melnyk et al., 2000; Stead et al., 2000). Overall, due to a lack of data on the hepatic levels of these compounds, it is difficult to ascertain any definitive role for increased Ser in homocysteine regulation. In the one‐carbon cycle, tetrahydrofolate requires a methyl group from Ser to convert to 5,10‐methylene tetrahydrofolate. This reaction reduces the cofactor NADP to NADPH. Thus, Ser indirectly contributes to the generation of reducing equivalents (NADPH), required for multiple purposes, including fatty acid synthesis. Previous studies show that SAAR induces a futile lipid cycle, that is, an increase in fatty acid synthesis and degradation (Perrone et al., 2008). Thus, the higher serinogenesis might be purposed to meet the increased demand for NADPH required for fatty acid synthesis. Such a role for Ser has been recently reported (Zhang et al., 2021).

A unique feature of SAAR‐induced serinogenesis is that the oxaloacetate appears to be sourced from mitochondria, not from the cytosol, that is, an increase in Pepck‐M, but not Pepck‐C. While both isoforms can induce serinogenesis, the reason for the preferential upregulation of Pepck‐M is unclear (Lowry et al., 1987). A possible explanation is the presence of an amino acid response element in the *Pck2* promoter (Mendez‐Lucas et al., 2014). To the best of our knowledge, no such promoter has been reported for *Pck1*. Atf4, another transcription factor, binds the response element in *Pck2* and regulates amino acid metabolism (Yu et al., 2021). We and others previously demonstrated that unfolded protein response and the Perk‐eIf2α‐Atf4 pathway, activated by amino acid limitation, are also upregulated by the SAAR diet (Mendez‐Lucas et al., 2014; Nichenametla et al., 2018; Pettit et al., 2017). Altogether, these lines of evidence suggest that the preferential upregulation of Pepck‐M might be occurring to maintain mitochondrial protein homeostasis. This idea is consistent with the fact that, despite the lower cytosolic protein synthesis, the SAAR diet did not alter the mitochondrial protein synthesis rates (Nichenametla et al., 2018; Pettit et al., 2017). Serinogenesis might also contribute to the biosynthesis of nucleotides required for cellular proliferation, another anabolic process, suggesting that SAAR‐induced changes in central carbon metabolism promote biomass accumulation, that is, growth. However, these biosynthetic processes deplete oxaloacetate from the TCA cycle, lowering the NADH production and ATP yield. Healthy organisms carefully balance the utilization of carbon and nitrogen sources between biomass accumulation (gluconeogenesis, nucleotide, and protein synthesis) and energy production (ATP yield) under nutrient limitation (Chen & Nielsen, 2019). Despite having ad libitum access and similar total amino acid content in SAAR, CR‐titrated, and CD diets, such adaptation is quite interesting. The only difference in these diets was the Cys concentrations or the availability of thiol groups. Changes in energy metabolism at the organismal level based on the thiol‐group abundance seem implausible. Yet, such adaptations occurred in yeast, where tRNA thiolation altered carbon and nitrogen homeostasis. Mutant tRNAs that cannot be thiolated resulted in an amino acid starvation phenotype despite sufficient availability of amino acids (Gupta et al., 2019). Overall, we propose that higher Pepck‐M induced by CR might be altering the metabolic pathways to sustain growth and survival under the limited availability of organic unsubstituted thiol (SH) groups at the cost of mitochondrial respiration.

Our study has some limitations. SAAR induced milder changes in adult mice than in young mice. SAA requirement for adult and old mice is much lower than for growing mice (Ishibashi & Kametaka, 1977). Hence, using the same concentration of Met (0.12%) in the SAAR diet for adult mice might not result in the same level of restriction as in young. Aging alters the transsulfuration pathway, which could affect the magnitude of CR and CR‐specific changes (Jeon et al., 2018). Future studies should try SAAR diets with less than 0.12% Met to increase the efficacy in adult mice. Although there is general agreement in the data from F344 rats and C57Bl6/J mice, both are inbred strains; these have limitations, including genetic homogeneity, idiosyncratic responses, and long‐term adaptation to the laboratory environment. Future studies must test the replicability of our findings in other inbred strains and outbred stocks. Plasma amino acid concentrations do not always reflect hepatic amino acid concentrations. Additional studies are required to determine hepatic amino acid concentrations, particularly cysteine, to understand SAAR effects clearly. Our proposed mechanism—an increase in serinogenesis at the cost of glyceroneogenesis—is based on transcriptional and translational changes in proteins and metabolites. Tracer‐based metabolic studies are essential to confirm this. Although outcomes from our epidemiological analysis and human feeding studies are congruent with the mechanisms observed in rodent studies, we cannot establish causality. Long‐term feeding studies with low‐SAA diets using metabolic tracers will help determine causality in humans.

## 4 METHODS

### 4.1 Animal and human studies designs

#### 4.1.1 Animal procedures and diets

All procedures were conducted according to the Institutional Animal Care and Use Committee guidelines of the Orentreich Foundation. Animals were singly housed and maintained on a 12:12 light–dark cycle at 20 ± 2°C and 50 ± 10% relative humidity. Food and acidified water were provided ad libitum. Food intake and body weight were recorded weekly. To find the discrete effects of MR (experiment 1) and CR (experiment 2), two cohorts of 8‐week‐old male F344 rats (Charles River Laboratories, Wilmington, MA, strain #002) were fed a Met‐titrated or Cys‐titrated diet for 12 weeks. In experiment 1, rats were randomly assigned to experimental diets (CD, SAAR, MR1, MR2, MR3, and MR4). Following an overnight fast, the rats were anesthetized with isoflurane, and blood was collected from the retro‐orbital plexus. Plasma obtained from blood (15,000 g/15 min/4°C) was stored at −80°C. Rats were sacrificed by CO_2_ asphyxiation and decapitation. Livers were snap‐frozen and held at −80°C. An aliquot of the liver was incubated overnight in RNA*later* (ThermoFisher Scientific, AM7021) at 4°C and transferred to 80°C the next day. The second rat cohort, obtained after analyzing data from the first cohort, was treated the same way as the first cohort except that the diets used were CD, SAAR, CR1, CR2, CR3, CR4, and CR5.

The effects of dietary fat content, sex, and age‐at‐onset on SAAR‐induced changes were determined by feeding three cohorts of C57BL/6J mice (Jackson Laboratory, stock #000664). The first (male, experiment 3) and second (female, experiment 4) cohorts of 8‐week‐old mice were fed CD and SAAR diets containing 10% Kcal and 60% Kcal fat for 16 weeks. The third cohort of male and female mice was aged in‐house until 18 months old (experiment 5) and fed CD and SAAR diets with 10% Kcal fat for 12 weeks. At the study's conclusion, blood and livers were obtained from 4‐hr fasted mice and were processed in the same manner as for rats. For all rodent experiments, the sample size was eight per group.

All diets fed to rats were isocaloric with the same total amino acid content (Table S1a,b; Research Diets). Amino acid content, in particular of Met and Cys, of all diets, was confirmed before starting the study (Table S9; Covance). All mouse diets were isocaloric and had same total amino acid content (Table S2). The CD and SAAR diets with 60% Kcal fat used for mice had similar energy densities, but it was different from that of the 10% Kcal fat diets. All diets were stored at −20°C during the study period.

#### 4.1.2 Epidemiological study design and subject selection

The population used for the current study was a subset of the parent study, which consisted of 278 coronary artery disease patients and 591 controls. Parent study objectives and subject recruitment details are described elsewhere (Janosikova et al., 2003). In the current cross‐sectional study, a subset of subjects (59 patients and 250 controls) was randomly selected and divided into six groups based on the number of MetS criteria they met, that is, from 0 to 5. The ratios of controls to patients and males to females as observed in the original study were maintained in all six groups. Met, tCys, and triglyceride concentrations were quantified in the baseline plasma from all 309 subjects. Ser was quantified from 287 subjects. Two patients were excluded due to severe hypertriglyceridemia (TAG >10 mmol/L), pancreatitis, and liver disease. Final data from 307 individuals were available for analysis.

#### 4.1.3 Controlled‐feeding study design and dietary formulation

Study subject recruitment details were published previously (Olsen et al., 2018, 2021). In brief, two randomized, controlled, double‐blind pilot studies were performed from 2016 to 2018 at the Centre for Clinical Nutrition at the Institute of Basic Medical Sciences, University of Oslo, Norway. Study 1, in which only SAA content was altered, was performed in overweight and obese women. In Study 2, SAA and fatty acid composition were altered in normal‐weight men and women. In both studies, diets were designed following the Nordic Nutrition Recommendations to meet percent energy requirements for macronutrients and to be isocaloric across the intervention groups (*Nordic Nutrition Recommendations 2012: Integrating nutrition and physical activity*, 2014). Diets were designed with fixed amounts of SAA per day to stay around the estimated average requirements in mg/kg body weight/day for each subject. Sample sizes and dietary compositions for both studies are described in Table S7.

### 4.2 Amino acid analyses

Plasma‐free amino acid levels in rats were determined by reverse‐phase HPLC‐UV. Separation chemistry is described in detail elsewhere (Bidlingmeyer et al., 1984; Heinrikson & Meredith, 1984). Drift was monitored by running standards between every ten samples. Rat hepatic Ser was quantified using a fluorometric DL‐Serine Assay Kit following the manufacturer's instructions (Biovision, catalog #K743). Values were expressed as nmol/mg tissue. Plasma tCys in the epidemiological study was quantified as described earlier (Krijt et al., 2001). Plasma Met and Ser in the epidemiological study were determined by LC–MS/MS using a commercially available kit for amino acid analysis (EZ:faast™, Phenomenex). As reported previously, plasma Ser from both human feeding studies was quantified by LC–MS/MS (Antoniades et al., 2009; Olsen et al., 2020, 2021). All plasma amino acid concentrations were expressed as μM.

### 4.3 Glycerol‐3‐phosphate

G3P was assayed in rat livers using a fluorometric kit following the manufacturer's recommendation (BioVision, catalog #K196). Unlike the manufacturer's instructions, we could not collect and quantify all the filtrate from the spin column. Hence, concentrations were expressed as the pmol/uL filtrate rather than the pmol/mg liver.

### 4.4 Glutathione

Non‐protein‐bound tGSH in the liver was determined, as described earlier, by an enzymatic recycling method using Ellman's reagent (Nichenametla et al., 2018). Values were expressed as μg/mg liver.

### 4.5 Transcriptional changes

Transcriptional changes were quantified by real‐time PCR of genes of interest and the housekeeping gene β2‐microglobulin. Detailed methods are provided elsewhere (Nichenametla et al., 2018). Inventoried assays for target genes were selected such that the primers span exon‐exon junctions (Table S10b, ThermoFisher Scientific). Fold‐change in mRNA expression of each diet group was expressed after normalizing with that of the CD group.

### 4.6 Translational changes

#### 4.6.1 ELISAs

Changes in protein expression were determined using ELISA (plasma proteins) and western blots (tissue proteins). Growth factors (Igf1 and Fgf21) and adipokines (leptin and adiponectin) were determined by quantitative sandwich ELISAs following the manufacturer's recommendation (Table S10a, Quantikine ELISA kits, R&D Systems Inc.) and expressed either as pg/mg or ng/mg of plasma protein. If samples were run on multiple plates, a pooled sample was run on all plates.

#### 4.6.2 Western blots

Tissues were homogenized in ice‐cold RIPA buffer, and proteins were probed by Western blots as described earlier (Nichenametla et al., 2018). Either β‐actin or vinculin was used as the loading control. To account for technical variation between gels, a batch control was run on all gels. The fold‐change in target proteins was expressed as a relative ratio to the loading controls. The sample size for each diet group ranged from 4 to 8. If membranes were reused to quantify a second target protein, they were stripped using Restore™ PLUS Western Blot Stripping Buffer (ThermoFisher Scientific, catalog #46430). Antibody sources and incubation conditions are presented in Table S10b.

### 4.7 Plasma glucose

Fasting (16 h in rats and 4 h in mice) blood glucose in rats and mice was determined from decapitated and retro‐orbital blood, respectively, using FreeStyle Lite® glucometers (Abbott Laboratories) and expressed as mg/dl.

### 4.8 Plasma triglycerides

Plasma triglycerides were quantified in microplates using Infinity Triglycerides Liquid Stable Reagent (ThermoFisher Scientific, catalog #TR22421) and a Triglycerides Standard from Pointe Scientific (T7531‐STD). Triglycerides Reagent (200 μl) was added to wells containing 10 μl plasma and incubated at 37°C for 5 min. Absorbance (500 nm) was measured and extrapolated to a standard curve. Triglyceride concentrations were expressed as mg/dl.

### 4.9 Statistics

Mean differences between CD and SAAR in the two rat cohorts were assessed using the two‐tailed Student's *t* test. For quantitative data, CD and SAAR groups from the two cohorts were combined to increase the statistical power and for easier graphical representation; however, semi‐quantitative data such as mRNA and protein expression from the two cohorts were tested individually and represented in two different panels. Data were analyzed in R (v 4.1.0) and GraphPad Prism. A *p*‐value ≤0.05 was considered statistically significant in all analyses.

#### 4.9.1 Linear mixed effect model

Linear mixed‐effect regression was used to test if there were any significant differences in the mean for each of the MR and the CR groups with respect to SAAR. Separate regressions were run for MR and CR diets. A random intercept was added to models to consider the heterogeneous effects contributed by each sample. *p*‐Values were adjusted for multiple comparison errors using false discovery rate (FDR).

#### 4.9.2 Trend tests

Two sets of estimated mean differences were extracted from the linear mixed models, namely MR groups versus SAAR and CR groups versus SAAR. Assuming a linear trend, two simple linear regressions were fitted for each of the two sets. We tested for any significant difference between the two estimated slopes using the Welch two‐sample *t* test (without the assumption of homogeneity).

#### 4.9.3 Equivalence tests

To be more rigorous in declaring the null hypothesis of equality is accepted (contrary to declaring the alternative cannot be rejected), equivalence tests in the form of two‐one‐sided *t* tests were conducted (Lakens, 2017). Following the usual practice, the smallest effect size of interest is set at 30% to characterize the upper and lower equivalence bounds. All raw *p*‐values from equivalence were corrected for multiple comparison errors by FDR corrections.

#### 4.9.4 Two‐way ANOVA

Data from mouse models were analyzed by two‐way ANOVA followed by Sidak's multiple comparison test. mRNA expression was considered significantly different from SAAR only if the fold‐change was >2 and *p*‐values were <0.05. *p*‐Values for interaction between the two independent variables were represented as μ_int_, and pairwise comparisons were represented by μ.

#### 4.9.5 Clinical and epidemiological data

Data from the epidemiological study were log‐transformed due to non‐normal distribution. The association of Met, tCys, and Ser with triglycerides was investigated with and without adjusting for age, sex, and BMI (log[triglycerides] ~ log[amino acid] + age + sex + BMI). We also investigated whether plasma amino acids changed with the number of MetS criteria with and without adjusting for age and sex (log[amino acid] ~ MetS criteria + age + sex). BMI was not considered for the second regression analysis as MetS criteria include obesity. Estimates were expressed as a percentage increase in triglycerides per percentage increase in amino acids. Estimates from the second model were expressed as a percentage change in amino acids with an increase in one MetS criterion. All data were expressed as geometric means and geometric standard deviations. In controlled‐feeding studies, differences in plasma Ser between groups were analyzed using linear mixed model regression with Ser as the outcome and group, visit, and their interaction term (group × visit) as predictors. Models were adjusted for baseline differences.

## AUTHOR CONTRIBUTIONS

Conceptualization, original manuscript draft, data visualization, supervision, project administration: SNN; methodology: CT, HR, KJV, and TO; formal analysis: SNN, VM, JPR, CT, TO, MP, and JS; investigation: SNN, DALM, DC, VLM, WM, GPA, BO, NEB, HR, KJV, AKS, MP, JS, and VK; writing (reviewing and editing): all authors.

## CONFLICT OF INTEREST

No authors have any conflict of interest.

## Supporting information


Figure S1

Figure S2

Figure S3

Figure S4

Appendix S1

## Data Availability

All data that support the findings of this study are presented either in the main manuscript or in supporting information.
