# Supplementary figures and images for: Cysteine restriction‐specific effects of sulfur amino acid restriction on lipid metabolism
Source: Aging Cell. 2022 Nov 19;21(12):e13739. doi: 10.1111/acel.13739 (PMC9741510; doi:10.1111/acel.13739)

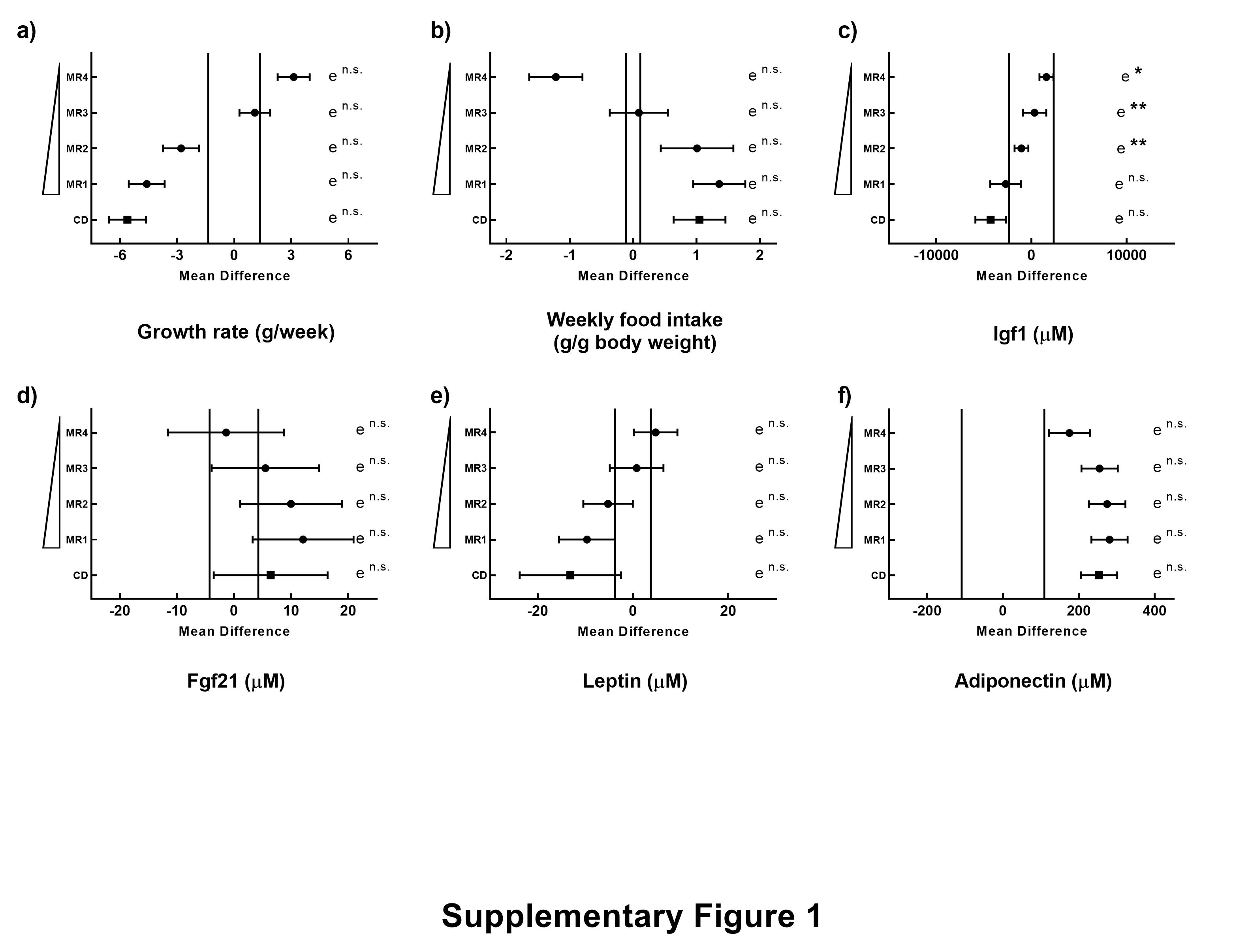

Supplement: Supplementary file 1 — Figure S1 [file ACEL-21-e13739-s004.jpg]

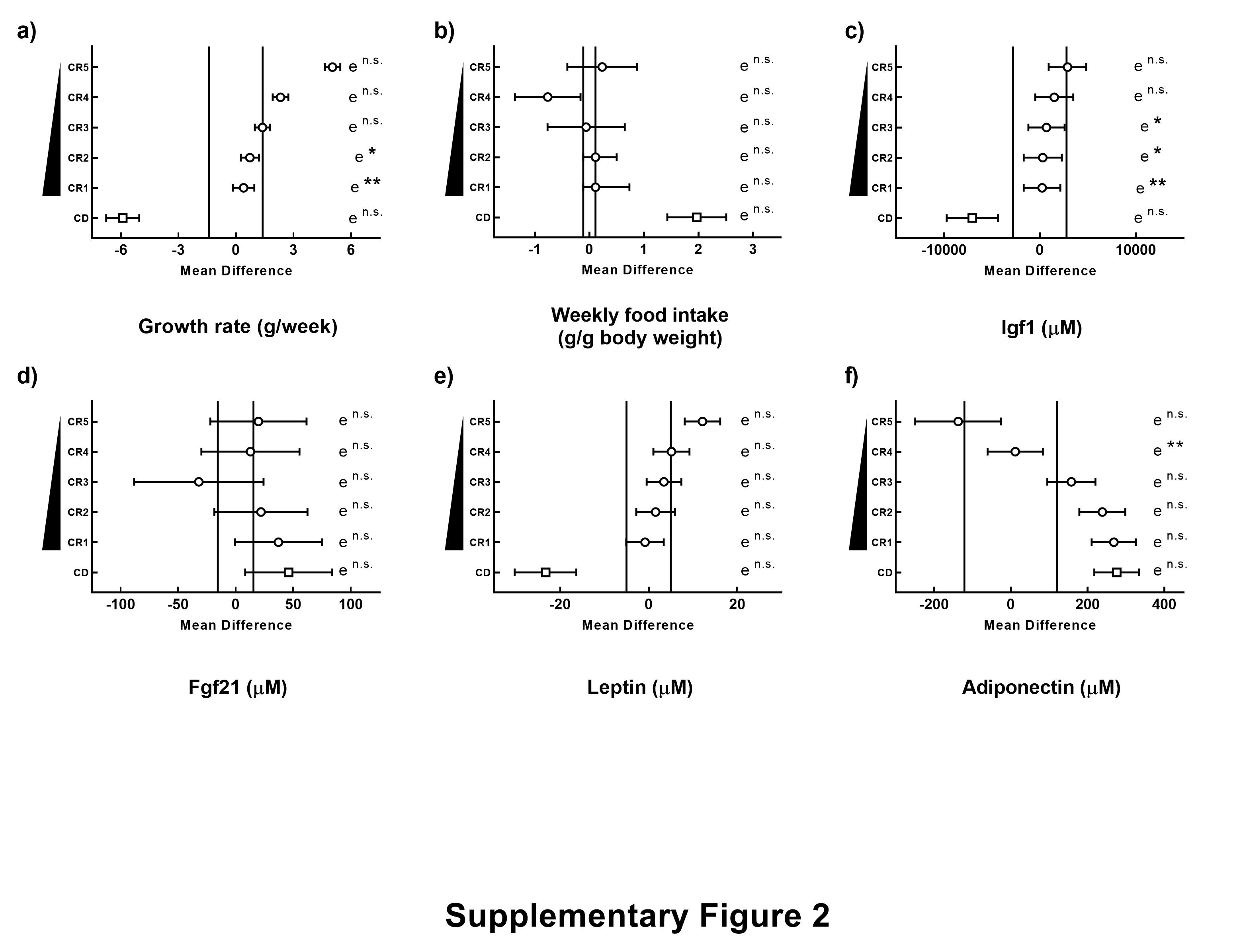

Supplement: Supplementary file 2 — Figure S2 [file ACEL-21-e13739-s002.jpg]

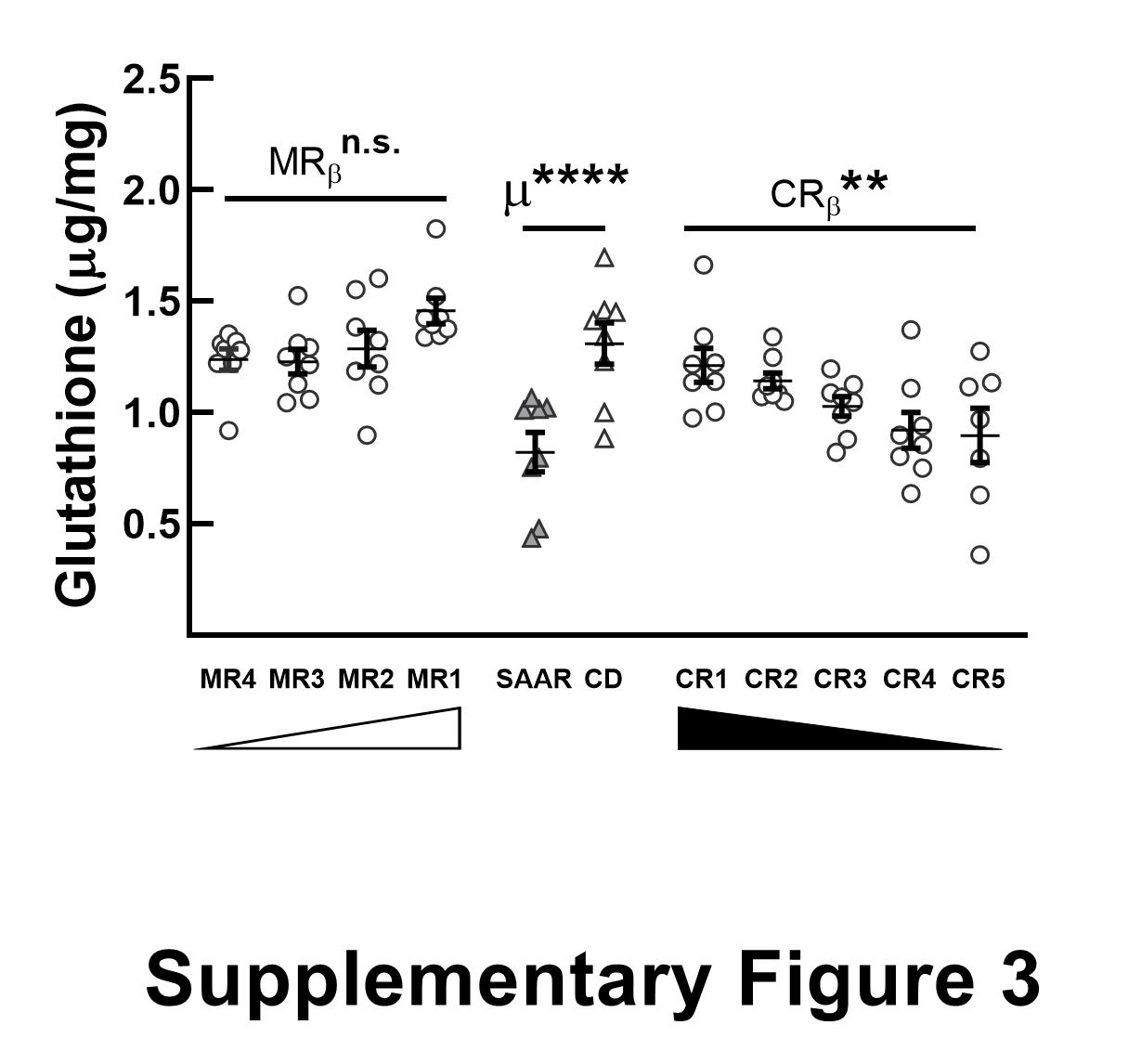

Supplement: Supplementary file 3 — Figure S3 [file ACEL-21-e13739-s005.jpg]

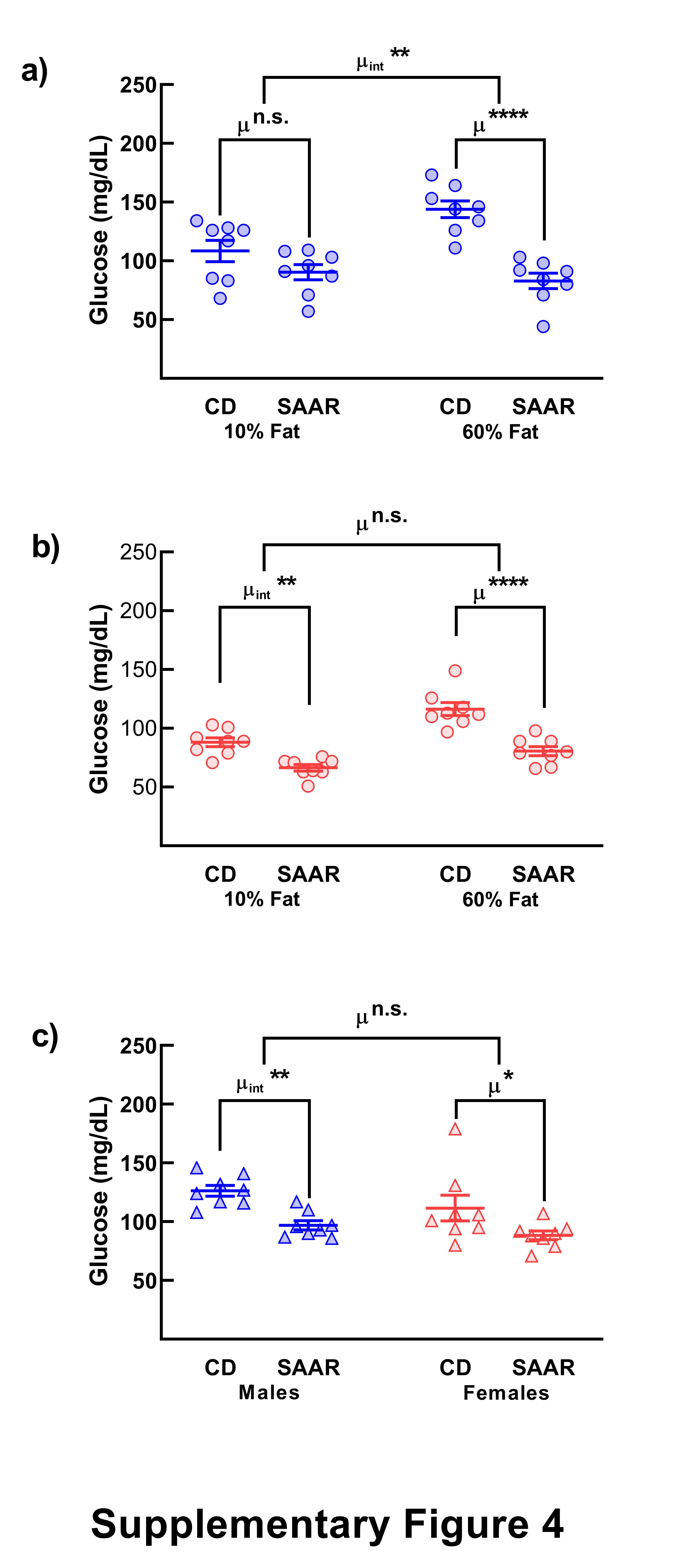

Supplement: Supplementary file 4 — Figure S4 [file ACEL-21-e13739-s001.jpg]
