# Supplementary material for: Cysteine restriction‐specific effects of sulfur amino acid restriction on lipid metabolism
Source: Aging Cell. 2022 Nov 19;21(12):e13739. doi: 10.1111/acel.13739 (PMC9741510; doi:10.1111/acel.13739)
Supplement: Supplementary file 5 — Appendix S1 [file ACEL-21-e13739-s003.docx]

| **Supplementary Table S1a. Composition of Diets Used in Rat Cohorts^a^** | | |
| --- | --- | --- |
| **Ingredient** |  | **Composition ((gm% (Kcal%))** |
|  |  |  |
| Protein |  | 14 (14) |
| Carbohydrate |  | 70 (69) |
| Fat |  | 8 (18) |
| Kcal/gm^b^ |  | 4.1 |
|  |  |  |
| L-Arginine |  | 1.12 (1.11) |
| L-Histidine-HCl-H_2_O |  | 0.33 (0.33) |
| L-Isoleucine |  | 0.82 (0.81) |
| L-Leucine |  | 1.11 (1.10) |
| L-Lysine |  | 1.44 (1.42) |
| L-Phenylalanine |  | 1.16 (1.15) |
| L-Threonine |  | 0.82 (0.81) |
| L-Tryptophan |  | 0.18 (0.18) |
| L-Valine |  | 0.82 (0.81) |
| Glycine |  | 2.33 (2.30) |
| ***DL-Methionine+L-Cysteine+L-Glutamic Acid* ^c^** |  | ***0.35 (3.51)*** |
| Corn Starch |  | 36.11 (35.65) |
| Maltodextrin |  | 12.5 (12.34) |
| Sucrose |  | 20.0 (19.74) |
| Cellulose |  | 5.0 (0) |
| Corn Oil |  | 8.0 (17.77) |
| Mineral Mix S10001 |  | 3.5 (0) |
| Vitamin Mix V10001 |  | 1.00 (0.99) |
| Choline Bitrartrate |  | 2 (0) |
| **^a^** Nutrient composition in this table is common for all the diets used in rat cohorts  **^b^** Energy density was determined using Atwater factor system  **^c^** *See Table 1b for specific concentrations of Met, Cys, and Glu in individual diets* | | |

| **Supplementary Table S1b. Composition of Experimental Diets Used in Rat Experiments** | | | | | | | | |
| --- | --- | --- | --- | --- | --- | --- | --- | --- |
| **Diet** |  | **Research Diets (Catalog No.)** |  | **DL-Methionine (gm% (Kcal%))** |  | **L-Cysteine (gm% (Kcal%))** |  | **L-Glutamic Acid (gm% (Kcal%))** |
| CD |  | A15021902 |  | 0.86 (0.85) |  | 0 (0) |  | 2.7 (2.67) |
| SAAR |  | A15021901 |  | 0.17 (0.17) |  | 0 (0) |  | 3.39 (3.35) |
| MR1 |  | A15070801 |  | 0.17 (0.17) |  | 0.5 (0.49) |  | 2.89 (2.86) |
| MR2 |  | A15070802 |  | 0.10 (0.09) |  | 0.5 (0.49) |  | 2.96 (2.91) |
| MR3 |  | A15070803 |  | 0.07 (0.07) |  | 0.5 (0.49) |  | 2.99 (2.96) |
| MR4 |  | A15070804 |  | 0.05 (0.05) |  | 0.5 (0.49) |  | 3.01 (2.96) |
| CR1 |  | A15021903 |  | 0.07 (0.07) |  | 0.5 (0.49) |  | 2.99 (2.95) |
| CR2 |  | A15021904 |  | 0.07 (0.07) |  | 0.25 (0.24) |  | 3.24 (3.20) |
| CR3 |  | A15021905 |  | 0.07 (0.07) |  | 0.12 (0.12) |  | 3.37 (3.33) |
| CR4 |  | A15021906 |  | 0.07 (0.07) |  | 0.06 (0.06) |  | 3.43 (3.38) |
| CR5 |  | A15021907 |  | 0.07 (0.07) |  | 0.03 (0.03) |  | 3.46 (3.42) |

| **Supplementary Table S2. Composition of Diets Used in Mouse Cohorts** | | | | | | | |
| --- | --- | --- | --- | --- | --- | --- | --- |
| **Ingredient** | **Composition in 10% Kcal**  **fat diets (gm%[kCal%])** | | |  | **Composition in 60% Kcal**  **fat diets (gm%[kCal%])** | | |
|  | **CD** |  | **SAAR** |  | **CD** |  | **SAAR** |
|  | **A14040402^a^** |  | **A14040401^a^** |  | **A14032002^a^** |  | **A14032001^a^** |
| Protein | 13 (14) |  | 13 (14) |  | 17 (13) |  | 17 (13) |
| Carbohydrate | 74 (76) |  | 74 (76) |  | 36 (28) |  | 36 (28) |
| Fat | 4 (10) |  | 4 (10) |  | 35 (60) |  | 35 (60) |
| Kcal/gm^b^ | 3.9 |  | 3.9 |  | 5.3 |  | 5.3 |
|  |  |  |  |  |  |  |  |
| L-Arginine | 1.09 (1.12) |  | 1.09 (1.12) |  | 1.48 (1.13) |  | 1.48 (1.13) |
| L-Histidine-HCl-H_2_O | 0.32 (0.32) |  | 0.32 (0.32) |  | 0.44 (0.33) |  | 0.44 (0.33) |
| L-Isoleucine | 0.8 (0.82) |  | 0.8 (0.82) |  | 1.09 (0.83) |  | 1.09 (0.83) |
| L-Leucine | 1.08 (1.1) |  | 1.08 (1.1) |  | 1.47 (1.1) |  | 1.47 (1.1) |
| L-Lysine | 1.4 (1.45) |  | 1.4 (1.45) |  | 1.91 (1.45) |  | 1.91 (1.45) |
| **DL-Methionine** | **0.86 (0.87)** |  | **0.12 (0.12)** |  | **0.86 (0.65)** |  | **0.12 (0.1)** |
| **L-Cysteine** | **0 (0)** |  | **0 (0)** |  | **0 (0)** |  | **0 (0)** |
| L-Phenylalanine | 1.13 (1.15) |  | 1.13 (1.15) |  | 1.53 (1.15) |  | 1.53 (1.15) |
| L-Threonine | 0.8 (0.82) |  | 0.8 (0.82) |  | 1.09 (0.83) |  | 1.09 (0.83) |
| L-Tryptophan | 0.17 (0.17) |  | 0.17 (0.17) |  | 0.24 (0.18) |  | 0.24 (0.18) |
| L-Valine | 0.8 (0.82) |  | 0.8 (0.82) |  | 1.09 (0.83) |  | 1.09 (0.83) |
| **L-Glutamic Acid** | **2.7 (2.77)** |  | **3.44 (3.55)** |  | **2.7 (2.05)** |  | **3.43 (2.6)** |
| Glycine | 2.26 (2.32) |  | 2.26 (2.32) |  | 3.08 (2.33) |  | 3.08 (2.33) |
| **Corn Starch** | **41.17 (42.41)** |  | **41.17 (42.41)** |  | **0 (0)** |  | **0 (0)** |
| **Maltodextrin** | **12.12 (12.49)** |  | **12.12 (12.49)** |  | **8.68 (6.55)** |  | **8.68 (6.55)** |
| Dextrose | 4.85 (5) |  | 4.85 (5) |  | 6.62 (5) |  | 6.62 (5) |
| Sucrose | 14.55 (14.99) |  | 14.55 (14.99) |  | 19.85 (15) |  | 19.85 (15) |
| Cellulose | 4.85 (0) |  | 4.85 (0) |  | 6.62 (0) |  | 6.62 (0) |
| **Lard** | **0 (0)** |  | **0 (0)** |  | **28.98 (49.28)** |  | **28.98 (49.28)** |
| Corn Oil | 4.46 (10.34) |  | 4.46 (10.34) |  | 6.09 (10.35) |  | 6.09 (10.35) |
| Mineral Mix S10001 | 3.39 (0) |  | 3.39 (0) |  | 4.63 (0) |  | 4.63 (0) |
| Vitamin Mix V10001 | 0.97 (1) |  | 0.97 (1) |  | 1.32 (1) |  | 1.32 (1) |
| Choline Bitrartrate | 0.19 (0) |  | 0.19 (0) |  | 0.26 (0) |  | 0.26 (0) |
| ^a^ Research Diets catalog number  ^b^ Energy density was determined using Atwater factor system | | | | | | | |

| **Supplementary Table S3. Effect of Sex on the Molecular Mechanisms of SAAR-induced Changes in Lipid Metabolism** | | | | | | | |
| --- | --- | --- | --- | --- | --- | --- | --- |
|  | 10% Kcal Fat Diets | | |  | 60% Kcal Fat Diets | | |
|  | Fold Change in Males (SAAR/CD) | Fold Change in Females (SAAR/CD) | Ratio of Fold Change (Males/Females) |  | Fold Change in Males (SAAR/CD) | Fold Change in Females (SAAR/CD) | Ratio of Fold Change ( Males/Females ) |
| Decrease in Glutathione | 0.35 | 0.42 | 0.85**^n.s.^** |  | 0.17 | 0.26 | ***0.67****** |
| Increase in NRF2 | 3.65 | 3.97 | 0.92**^n.s.^** |  | 5.97 | 3.69 | ***1.62**** |
| Increase in PHGDH | 5.46 | 11.45 | 0.48**^n.s.^** |  | 11.56 | 5.06 | ***2.28**** |
| Increase in PCK2 | 24.55 | 23.68 | 1.04**^n.s.^** |  | 100.15 | 96.54 | 1.04**^n.s.^** |
| Decrease in Triglycerides | 0.87 | 0.89 | 0.97**^n.s.^** |  | 0.69 | 0.71 | 0.98**^n.s.^** |
| Decrease in Perigonadal adipose depot weight | 0.67 | 1.00 | ***0.67****** |  | 0.44 | 0.96 | ***0.46****** |
| Note: Experiments 3, 4, and 5 (for details see section on methods) were conducted at different time points but under the same animal husbandry conditions, with similar diets, and in the same animal facility. For data analysis purposes, all three experiments were considered as one. Fold-changes and asterisks in columns 3 and 6 represent μ_int_-values obtained from two-way ANOVA, considering diet and sex as independent variables. | | | | | | | |

| **Supplementary Table S4. Effect of Age-at-onset on the Molecular Mechanisms of SAAR-induced Changes in Lipid Metabolism** | | | | | | | |
| --- | --- | --- | --- | --- | --- | --- | --- |
|  | Males | | |  | Females | | |
|  | Fold Change in Young (SAAR/CD) | Fold Change in Adult  (SAAR/CD) | Ratio of Fold Change (Y/A) |  | Fold Change in Young (SAAR/CD) | Fold Change in Adult (SAAR/CD) | Ratio of Fold Change (Y/A) |
| Decrease in Glutathione | 0.35 | 0.43 | 0.82**^n.s.^** |  | 0.42 | 0.71 | **0.59***** |
| Increase in NRF2 | 3.65 | 2.61 | 1.40**^n.s.^** |  | 3.97 | 1.36 | **2.92****** |
| Increase in PHGDH | 5.46 | 4.34 | 1.26**^n.s.^** |  | 11.45 | 4.87 | 2.35**^n.s.^** |
| Increase in PEPCK-M | 24.55 | 55.00 | 0.45**^n.s.^** |  | 23.68 | 7.75 | **3.05******* |
| Decrease in Triglycerides | 0.87 | 0.87 | 1.00**^n.s.^** |  | 0.89 | 0.99 | 0.90**^n.s.^** |
| Decrease in Perigonadal adipose depot weight | 0.67 | 0.68 | 1.00**^n.s.^** |  | 1.00 | 0.64 | 1.58**^n.s.^** |
| Note: Experiments 3, 4, and 5 (for details, see the section on methods) were conducted at different time points but under the same animal husbandry conditions, with similar diets, and in the same animal facility. For data analysis purposes, all three experiments were considered as one. Fold-changes and asterisks in columns 3 and 6 represent μ_int_-values obtained from two-way ANOVA, considering diet and age-at-onset as independent variables. | | | | | | | |

| **Supplementary Table S5: Epidemiological Study Population Characteristics** | | | | | | | | |
| --- | --- | --- | --- | --- | --- | --- | --- | --- |
| **Characteristic** | **0 MetS criteria** | **1 MetS criteria** | **2 MetS criteria** | **3 MetS criteria** | **4 MetS criteria** | **5 MetS criteria** | **P_trend_** |  |
| Sample size (n) | 50 | 50 | 50 | 50 | 50 | 57 | - |  |
| Age (y) | 38.5 (1.42) | 47.1 (1.34) | 51.3 (1.17) | 52.9 (1.16) | 53.4 (1.16) | 54.1 (1.11) | < 0.001 |  |
| Males, n (%) | 16 (32) | 23 (46) | 30 (60) | 37 (74) | 38 (76) | 41 (71.9) | < 0.001 |  |
| BMI (kg/m^2^) | 22 (1.09) | 24.6 (1.14) | 26.3 (1.11) | 28.3 (1.14) | 29.2 (1.15) | 30.6 (1.14) | < 0.001 |  |
| Triacylglycerols  (mmol/l) | 0.89 (1.38) | 1.01 (1.45) | 1.18 (1.45) | 1.83 (1.43) | 2.34 (1.39) | 3.17 (1.54) | < 0.001 |  |
| CAD patient, n (%) | 2 (4) | 8 (16) | 15 (30) | 24 (48) | 28 (56) | 41 (71.9) | < 0.001 |  |
| Smoker, n (%) | 13 (26) | 12 (24) | 6 (12) | 13 (26) | 5 (10) | 6 (10.5) | < 0.001 |  |
| Former smoker, n (%) | 8 (16) | 10 (20) | 17 (34) | 21 (42) | 31 (62) | 41 (71.9) | < 0.001 |  |
| Note: Continuous variables are presented as geometric mean (geometric standard deviation). Categorical variables are presented as n (%). | | | | | | | | |

| **Supplementary Table S6: Associations of Plasma Amino Acids with Triglycerides and MetS Criteria** | | | | | |
| --- | --- | --- | --- | --- | --- |
| 1. Plasma amino acids and triglycerides | | | | | |
|  | n | Unadjusted model | | Adjusted model^a^ | |
| Amino Acids | | Estimate^b^ (95 % CI) | p-value | Estimate^b^ (95 % CI) | p-value |
| Met | 307 | -0.001 (-0.281,0.279) | 0.994 | -0.066 (-0.3,0.168) | 0.578 |
| tCys | 307 | 1.338 (0.997,1.779) | < 0.001 | 0.546 (0.125,0.968) | 0.011 |
| Ser | 287 | -0.397 (-0.619,-0.174) | 0.001 | -0.302 (-0.496,-0.108) | 0.002 |
| tCys/Ser | 287 | 0.57 (0.37, 0.769) | < 0.001 | 0.337 (0.155, 0.52) | < 0.001 |
| 1. Plasma amino acids and MetS criteria | | | | | |
|  | | Unadjusted model | | Adjusted model^c^ | |
| Amino Acids | | β-estimate^d^ (confidence intervals) | p-value | β-estimate^d^ (confidence intervals) | p-value |
| Met | 307 | 0.939 (-0.615,2.518) | 0.237 | 0.881 (-0.883,2.677) | 0.329 |
| tCys | 307 | 4.104 (3.143,5.075) | < 0.001 | 2.154 (1.18,3.137) | < 0.001 |
| Ser | 287 | -2.145 (-4.123,-0.127) | 0.037 | -2.563 (-4.729,-0.348) | 0.024 |
| tCys/Ser | 287 | 5.306 (3.08,7.579) | < 0.001 | 4.294 (1.862,6.785) | < 0.001 |
| ^a^ Adjusted for age, sex, and BMI  ^b^ β-estimates represent % change in triglycerides per 1 % change in the amino acid concentration.  ^c^ Adjusted for age and sex  ^d^ β-estimates represent % change in the amino acid concentration per increase in each MetS criteria | | | | | |

| **Supplementary Table S7. Dietary Sulfur Amino Acid and Fat Composition in Short-term Feeding Studies** | | | | | | |
| --- | --- | --- | --- | --- | --- | --- |
|  | SAA intake ^a^ (g/day(mg/Kg BW/day)) | | Fat intake (% total energy) | | Sample size | |
|  | Men | Women | PUFA | SFA | Males | Females |
| Study 1 | | | | | | |
| SAA_low_ | -- | 1.5 (17.7) | 7.2 | 3.9 | 0 | 7 |
| SAA_hi_ | -- | 5.5 (71.5) | 7.2 | 3.9 | 0 | 6 |
| Study 2 | | | | | | |
| SAA_low+PUFA_ | 1.19 (15.3) | 0.93 (14.2) | 10 | 5.5 | 2 | 5 |
| SAA_hi+SFA_ | 6.01 (82.0) | 5.75 (87.9) | 3.3 | 13.5 | 2 | 5 |
| ^a^ represents the intake of Met and Cys together | | | | | | |

| **Supplementary Table S8. Changes in Plasma Ser in Short-term Controlled Feeding Studies** | | | | |
| --- | --- | --- | --- | --- |
| Diet | Plasma Ser (mmol/L, Mean±SEM) | | | p-value for time-related (day 0-7) changes between the diet groups |
|  | Day 0 | Day 3 | Day 7 |  |
|  |  |  |  |  |
| SAA_low_ | 100.85±9.23 | 102.79±6.84 | 96.89±7.72 | 0.94 |
| SAA_hi_ | 103.70±6.81 | 107.07±6.64 | 100.72±9.97 |  |
|  |  |  |  |  |
| SAA_low+PUFA_ | 105.82±8.06 | 105.24±6.42 | 118.02±8.49 | 0.018 |
| SAA_hi+SFA_ | 101.21±8.55 | 91.96±7.47 | 96.12±6.59 |  |

| **Supplementary Table S9. Confirmation of Sulfur Amino Acid Concentration in Diets Used for Rat Cohorts** | | | | |
| --- | --- | --- | --- | --- |
| Diet |  | Methionine (Detected/Expected) |  | Cysteine (Detected/Expected) |
| CD |  | **0.783**/0.86 |  | **<0.01**/0 |
| SAAR |  | **0.162**/0.17 |  | **<0.01**/0 |
| MR1 |  | **0.164**/0.17 |  | **0.33**/0.5 |
| MR2 |  | **0.109**/0.1 |  | **0.36**/0.5 |
| MR3 |  | **0.071**/0.07 |  | **0.337**/0.5 |
| MR4 |  | **0.056**/0.05 |  | **0.348**/0.5 |
| CR1 |  | **0.075**/0.07 |  | **0.338**/0.5 |
| CR2 |  | **0.073**/0.07 |  | **0.178**/0.25 |
| CR3 |  | **0.079**/0.07 |  | **0.101**/0.125 |
| CR4 |  | **0.075**/0.07 |  | **0.051**/0.062 |
| CR5 |  | **0.073**/0.07 |  | **0.033**/0.031 |
| Note: Dietary analysis was performed at Covance, Madison, WI | | | | |

| **Supplementary Table S10. Experimental Details of Protein and mRNA Quantification** | | | | | | | | | |
| --- | --- | --- | --- | --- | --- | --- | --- | --- | --- |
| 1. Enzyme-linked immunosorbent assays | | | | | | | | | |
| Plasma marker | | Vendor | | | Catalog# | | Comments | | |
| Igf-1 | | R&D Systems | | | MG100 | | Mouse/Rat Kit | | |
| Adiponectin | | R&D Systems | | | RRP300 | | Rat Kit | | |
| Fgf21 | | R&D Systems | | | RRP300 | | Rat Kit | | |
| Leptin | | R&D Systems | | | MOB00 | | Mouse/Rat Kit | | |
| 1. Western blot details | | | | | | | | | |
| *Protein of interest* | *Antibody source* | | | | *Dilution* | | | *Incubation conditions* | |
|  | *Primary* | | | *Secondary* | *Primary* | *Secondary* | | *Primary* | *Secondary* |
| Phgdh (Rat) | Cell Signaling  13428 | | | Cell Signaling  7074 | 1:1000 | 1:7500 | | 5% milk O/N @ 4^o^C | 5% milk 1 hr @ RT |
| Phgdh (Mouse) | Cell Signaling  13428 | | | Cell Signaling  7074 | 1:1000 | 1:7500 | | 5% milk O/N @ 4^o^C | 5% milk 1 hr @ RT |
| Pepck-M (Rat) | Cell Signaling  6924 | | | Cell Signaling  7074 | 1:1000 | 1:7500 | | 5% milk O/N @ 4^o^C | 5% milk 1 hr @ RT |
| Pepck-M (Mouse) | Cell Signaling  6924 | | | Cell Signaling  7074 | 1:1000 | 1:7500 | | 5% milk O/N @ 4^o^C | 5% milk 1 hr @ RT |
| Nrf2 (Mouse) | Cell Signaling  20733 | | | Cell Signaling  7074 | 1:1000 | 1:7500 | | 5% milk O/N @ 4^o^C | 5% milk 1 hr @ RT |
| β-Actin | MilliporeSigma  A5441 | | | Bio-Rad  1706516 | 1:20000 | 1:20000 | | 5% milk 30 min @ RT | 5% milk 30 min @ RT |
| Vinculin | Proteintech  66305-1-lg | | | Bio-Rad  1706516 | 1:10000 | 1:20000 | | 5% milk 30 min @ RT | 5% milk 30 min @ RT |
| c) Assay details for mRNA quantification | | | | | | | | | |
| *Gene of interest* | | | *TaqMan assay ID* | | | | | | |
| *Phgdh* | | | Rn01534200_g1 | | | | | | |
| *Pck1* | | | Rn01529014_m1 | | | | | | |
| *Pck2* | | | Rn03648110_m1 | | | | | | |
| *G6pc* | | | Rn00689876_m1 | | | | | | |
| *β2M* | | | Rn00560865_m1 | | | | | | |

**Supplementary Figures S1 and S2:** Equivalence tests (two one-sided t-tests) were conducted to find which of the Met-titrated (MR1, MR2, MR3, and MR4) and Cys-titrated (CR1, CR2, CR3, CR4, and CR5) diets resulted in an equivalent response as the SAAR diet, in each of the six phenotypes tested (body weight, food intake, Igf1, Fgf21, leptin, and adiponectin). The X-axis represents the mean difference of each diet (CD [■ or □], MR [●] and CR [○] diets) from the SAAR diet, horizontal lines on either side of the symbols represent 95% confidence intervals, vertical lines represent the 30% confidence bounds, and the asterisks represent the p-values obtained from equivalence tests. Note: Although we did not get a significant p-value for growth rate, food intake, and Fgf21, we considered MR3 as the equivalent dose because it falls within the 30% confidence bounds (vertical lines) for most of the phenotypes. White and black triangles on the y-axis represent the SAA concentration gradient in MR and CR diets, respectively.

**Supplementary Figure S3: SAAR-induced depletion of hepatic glutathione is CR-specific.** Hepatic glutathione levels were quantified from F344 rats after 12 weeks on MR- and CR- titrated diets. CR, but not MR, induced a dose-dependent decrease in hepatic glutathione. *Note*: For statistics and annotations refer to Figure 1. n = 8/group. White and black triangles on the x-axis represent the SAA concentration gradient in MR and CR diets, respectively.

**Supplementary Figure S4: Blood glucose concentrations in mouse models.** Blood glucose was measured in 4h-fasted a) young (8-week-old) male and b) female mice on 10% and 60% Kcal fat diets and in c) adult (18-month-old) male and female mice on 10% Kcal fat CD and SAAR diets. Except in male mice on 10% Kcal fat, SAAR decreased blood glucose levels in all models. *Note*: For statistics and annotations refer to Figure 5; n = 8/group.
